# Repeated Emergence of Variant TetR Family Regulator, FarR, and Increased Resistance to Antimicrobial Unsaturated Fatty Acid among Clonal Complex 5 Methicillin-Resistant Staphylococcus aureus

**DOI:** 10.1128/aac.00749-22

**Published:** 2023-02-06

**Authors:** Camryn M. Bonn, Iftekhar M. Rafiqullah, John A. Crawford, Yi Meng Qian, Jennifer L. Guthrie, Marta Matuszewska, D. Ashley Robinson, Martin J. McGavin

**Affiliations:** a Department of Microbiology, University of Western Ontario, London, Ontario, Canada; b Department of Cell and Molecular Biology, University of Mississippi Medical Center, Jackson, Mississippi, USA; c Schulich School of Medicine and Dentistry, University of Western Ontario, London, Ontario, Canada; d Department of Medicine, University of Cambridge, Cambridge, United Kingdom; e Center for Immunology and Microbial Research, University of Mississippi Medical Center, Jackson, Mississippi, USA

**Keywords:** MRSA, *Staphylococcus aureus*, TetR family regulator, efflux pumps, mechanisms of resistance

## Abstract

Resistance-nodulation-division (RND) superfamily efflux pumps promote antibiotic resistance in Gram-negative pathogens, but their role in Gram-positive pathogens, including methicillin-resistant Staphylococcus aureus (MRSA) is undocumented. However, recent *in vitro* selections for resistance of S. aureus to an antimicrobial fatty acid, linoleic acid, and an antibiotic, rhodomyrtone, identified H121Y and C116R substitution variants, respectively, in a TetR family regulator, FarR, promoting increased expression of the RND pump FarE. Hypothesizing that *in vivo* selection pressures have also promoted the emergence of FarR variants, we searched available genome data and found that strains with FarR^H121Y^ from human and bovine hosts have emerged sporadically in clonal complexes (CCs) CC1, CC30, CC8, CC22, and CC97, whereas multiple FarR variants have occurred within CC5 hospital-associated (HA)-MRSA. Of these, FarR^E160G^ and FarR^E93EE^ were exclusive to CC5, while FarR^C116Y^, FarR^P165L^, and FarR^G166D^ also occurred in nonrelated CCs, primarily from bovine hosts. Within CC5, FarR^C116Y^ and FarR^G166D^ strains were polyphyletic, each exhibiting two emergence events. FarR^C116Y^ and FarR^E160G^ were individually sufficient to confer increased expression of FarE and enhanced resistance to linoleic acid (LA). Isolates with FarR^E93EE^ were most closely related to S. aureus N315 MRSA and exhibited increased resistance independently of FarR^E93EE^. Accumulation of pseudogenes and additional polymorphisms in FarR^E93EE^ strains contributed to a multiresistance phenotype which included fosfomycin and fusidic acid resistance in addition to increased linoleic acid resistance. These findings underscore the remarkable adaptive capacity of CC5 MRSA, which includes the polyphyletic USA100 lineage of HA-MRSA that is endemic in the Western hemisphere and known for the acquisition of multiple resistance phenotypes.

## INTRODUCTION

Efflux pumps of the resistance-nodulation-division (RND) superfamily are well known for conferring vital resistance mechanisms toward antibiotics and chemotherapeutic agents (1). This efflux pump superfamily was discovered in Gram-negative enteric bacteria through mutations which conferred sensitivity to xenobiotic compounds such as acriflavine and acridine (2, 3), and it is now apparent that one of its major physiological functions is to facilitate colonization of the gut through efflux of bile salts or related detergents and microbial metabolites (4–8). Consequently, regulation can be complex and derives input from stress-related signals and multiple transcriptional activators (9). However, a common theme is their control by TetR family regulators (TFRs) which repress their expression in the absence of an inducing stimulus (10–12). Moreover, because RND efflux pumps can accommodate a range of structurally unrelated antimicrobial compounds, a paradigm of emergence of resistance during antimicrobial therapy of Gram-negative pathogens is attributed to mutations in the TFR repressor or its cognate DNA binding motif, leading to de-repression of the efflux pump (13–16).

This resistance paradigm is well-established in Gram-negative pathogens, but the functions of RND pumps and their contribution to the emergence of resistance is less well-defined in Gram-positive bacteria. Of particular interest, Staphylococcus aureus colonizes the nose and skin in approximately 30% of the human population but is also a leading cause of infectious morbidity and mortality and a significant threat to public health due to the emergence of resistance to multiple antimicrobial agents (17, 18). An important innate defense mechanism that deters colonization of S. aureus and other pathogens is antimicrobial unsaturated free fatty acids (uFFA), which bacteria are exposed to in secretions of the upper respiratory tract and sebaceous secretions of the skin (19, 20). Consistent with the paradigm for emergence of resistance through altered expression of RND pumps, we conducted *in vitro* selection for increased resistance to linoleic acid (LA) in the USA300 strain of community-acquired methicillin-resistant S. aureus (CA-MRSA), which led to the recovery of a H121Y variant in a previously uncharacterized TFR which we designated FarR; this led to enhanced resistance through increased expression of the divergently transcribed RND efflux pump FarE (21). Similarly, others have identified a FarR^C116R^ variant that confers resistance to the plant-derived antimicrobial rhodomyrtone (22).

In related work, S. aureus MRSA strain COL was subjected to *in vitro* selection for increased resistance to an oxadiazole antibiotic, representing a new class of non-β-lactam antibiotics which also target penicillin-binding proteins in Gram-positive bacteria (23, 24). This promoted a T172I substitution in SACOL2566 encoding efflux pump MmpL (23), which is identical to the efflux pump FarE (SAUSA300_2489) that we have described in S. aureus USA300. Consequently, *in vitro* selections for enhanced resistance of S. aureus to various antimicrobial agents have identified amino acid substitutions in FarR or in the FarE/MmpL efflux pump (21–23). In view of these considerations, and the notoriety of S. aureus in acquiring resistance to antibiotics, we hypothesized that *in vivo* exposure to either antimicrobial therapy or host-derived antimicrobial fatty acids could be a driving force in the emergence of strains with altered RND efflux pump expression. However, unlike other TFRs which primarily repress expression of the target efflux pump, FarE was not expressed in the absence of FarR (21), making it unlikely that enhanced resistance could be achieved through mutations that inactivate *farR* function or expression. Here, we report on our search for amino acid substitution variants in FarR across the spectrum of S. aureus sequenced genomes. We found that variant FarR proteins have repeatedly emerged in the CC5 lineage of health care-associated MRSA (HA-MRSA), of which FarR^C116Y^ and FarR^E160G^ are individually sufficient to promote increased resistance to LA.

## RESULTS

### Assessment of FarR variation in the context of S. aureus phylogenetic diversity.

**FIG 1:**
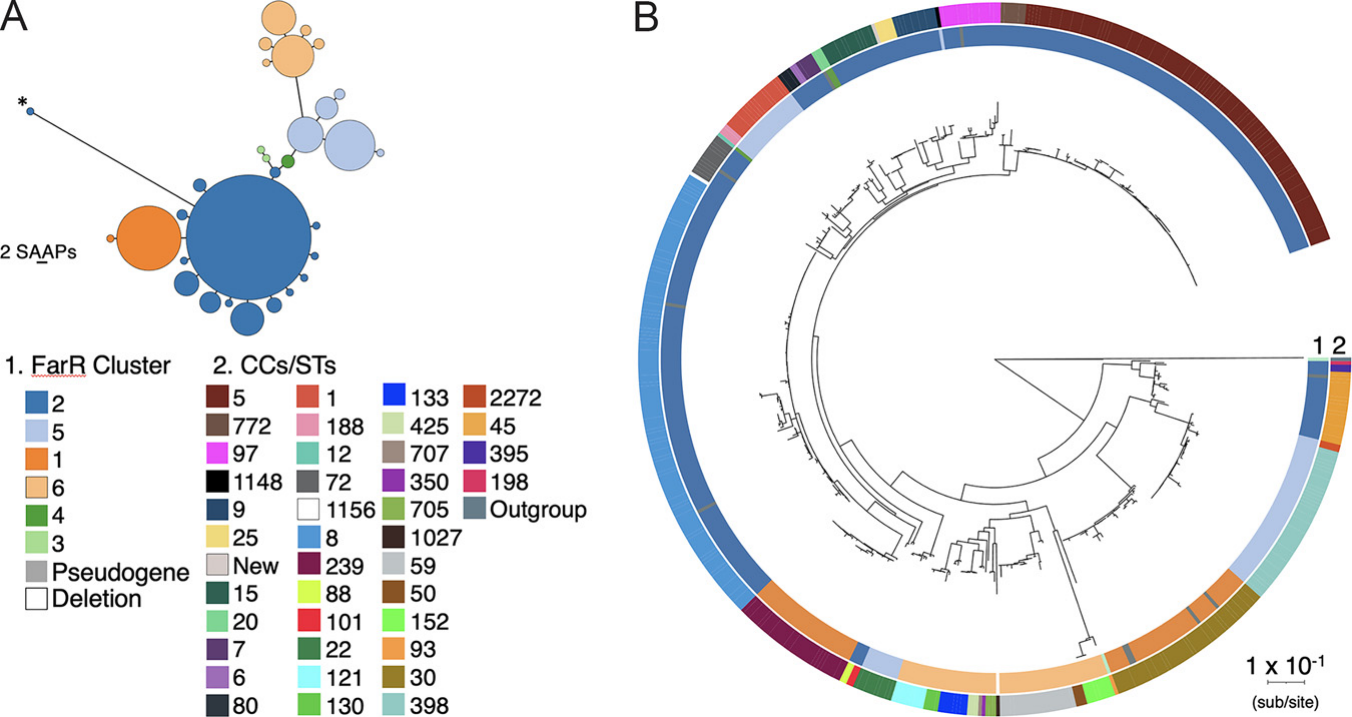
Grapes plot of six major FarR clusters and associated variants (A) and mapping of FarR clusters on a proteome based Staphylococcus aureus phylogenetic map (B). For the Grapes plot (A), the minimum spanning tree was constructed by alignment of 564 full-length FarR proteins using GrapeTree, which identified 6 primary clusters and associated variants. Points represent groups of identical elements, with point size correlated with number of elements on a log scale. Phylogenetic distance is scaled to two single-amino acid polymorphisms (2SAAP). The asterisk on cluster 2 (*) marks FarR variant 2c where duplication of an amino acid codon at E93 alters the alignment. For the phylogenetic map (B), conceptually translated nucleotide sequences available from PATRIC are shown for 574 S. aureus and 1 *S. argenteus* strains using PhyloPhlAn3. Initial phylogenetic analysis used FastTree, which was refined with RaxML. The six primary FarR clusters are mapped on ring 1, while clonal complex associations are mapped on ring 2. The colored legend for clonal complex designations is presented in the same order of appearance as on ring 2, beginning with CC5 and progressing in descending order in each column from left to right, ending with the *S. argenteus* outgroup.

We first assessed variation in FarR across all S. aureus genomes that were designated complete (as of 2020) in the Pathosystems Resource Information Center (PATRIC) database (25) (Table S1 in the supplemental material), which currently has data on 21,099 genomes. These 574 completed genomes yielded 563 full-length FarR proteins, for which multiple alignment revealed 28 variations that could be grouped into 6 primary clusters (Table 1 and Fig. S1). The FarR clusters exhibited a high degree of conservation, with the most diverse cluster, 6, exhibiting 88% identity and 95% similarity relative to cluster 1. The relatedness of the FarR clusters and proportion of strains associated with each are shown as a Grapes plot in Fig. 1A, and these clusters are mapped on a S. aureus phylogenetic tree constructed from conceptually translated proteomes of the 574 strains using PhyloPhlAn3 (Fig. 1B). Each variant was also used to match identical proteins in the NCBI database of S. aureus subsp. *aureus* proteins. The number of identical protein accessions for each variant and their clonal complex associations are reported in Table 1; for contextual purposes, the total number of genomes from the PATRIC database that correspond to specific multilocus sequence type (MLST) designations are provided as a footnote in Table 1. Eleven strains did not have full-length FarR proteins due to single-nucleotide deletions, transposon or IS element insertions, and, in the case of one strain, a deletion that spanned *farR* and most of the *farE* efflux pump (Fig. 1B, Table S1).

**TABLE 1:** Clonal complex association of six primary FarR clusters and associated variants^*a*^

FarR cluster	Primary sequence and variants^*b*^	CC(s)^*c*^	Protein accessions (*n*)^*d*^
1	MKETDLRVIKTKKALSSSLLQLLEQQLFQTITVNQICDNALVHRTTFYKHFYDKYDLLEYLFNQLTKDYFARDISDRLNHPFQTMSDTINNKEDLREIAEFQEEDAEFNKVLKNVCIKIMHNDIKNNRDRIDIDSDIPDNLIFYIYDSLIEGFIHWIKDEKIDWPGEDIDNIFHKVINIKIK	30, **239**	2,649
1a	D134Y	30	40
2	MKETDLRVIKTKKALSSSLLQLLEQQLFQTITVNQICDNALVHRTTFYKHFYDKYDLLEYLFNQLTKDYFARDISDRLNHPFQTMSDTINNKEDLREIAEFQEEDAEFNKVLKNVCIKIMHNDIKNNRDRIDIDSDIPDNLIFYIYDSLIEGFIHWIKDEKIDWPGEDIDNIFH**RL**INIKIK	5, 6, 7, 8, 9, 15, 25, 72, 80, 88, 97, 101, 772, 1,156	12,894
2a	E160G	5	82
2b	G166D	5, 8, 97	9
2c	E93EE	5	61
2d	V115I	5	3
2e	R96K	5	4
2f	M85I	**395**	11
2g	A14P	5 (ST228)	32
2h	S16L	5 (ST228)	2
2i	R77H	97	11
2j	R130H	8	6
2k	K110V	8	2
2l	S75G	**45, 198**	716
2m	L78I,N79T	97	1
2n	S17T	101	36
3	MKETDLRVIKTKKALSSSLLQLLEQQLFQTITVNQICDNALVHRTTFYKHFYDKYDLLEYLFNQLTKDYFARDISDRLNHPFQT**I**SDTINNKEDLREIAEFQEED**I**EFNKVLKNVCIKIMH**D**DIKNNRDRIDIDSD**V**PDNLIFYIYDSLIEGF**u**HWIKDEKIDWPGEDIDNIFH**RL**INIKIK	93	266
3a	I106S	Outgroup	125
4	MKETDLRVIKTKKALSSSLLQLLEQQLFQTITVNQICDNALVHRTTFYKHFYDKYDLLEYLFNQLTKDYFARDISDRLNHPFQT**I**SDTINNKEDLR**D**IAEFQEEDAEFNKVLKNVCIKIMHNDIKNNRDRIDIDSDIPDNLIFYIYDSLIEGF**M**HWIKDEKIDWPGEDIDNIFH**RL**INIKIK	12, 20	167
5	MKETDLRVIKTKKALSSSLLQLLEQQLFQTITVNQICDNALVHRTTFYKHFYDKYDLLEYLFNQLTKDYFARDISDRLNHPFQT**I**SDTINNKEDLR**D**IAEFQEEDAEFNKVLKNVCIKIMHNDIKNNRDRIDIDSDIPDNLIFYIYDSLIEGF**M**HWIKDEKIDWPGE**E**ID**K**IFHKVINIKIK	1, 188, **1,148**	1,128
5a	Q35L	**398, 2,272**	1,530
5b	Q35L, V115F	**398**	1
5c	D136H	**22**	2,440
5d	D136H, I142T	**22**	2
6	MKETDLRVIKTKKALSSSLLQLLEQ**H**LFQTITVNQIC**H**NALVHRTTFYKHFYDKYDLLEYLFNQLTK**A**YFA**T**DISDRLNHPFQT**IN**DTINNKEDL**QKV**A**D**FQ**Q**EDAEFNKVLKNVCIKIM**ND**DIKNN**S**DRID**V**D**G**DIP**N**NLLFYIYDSLIEGF**L**HWIKDEKIDWP**S**E**E**ID**K**IFHKVINIKIK	425	2
6a	D147G	50, 130, 133, 121, 152, 350	841
6b	D147G, H80L	133	2
6c	D147G, H155Y	705	48
6d	D147G, S128C	705, 707	89
6e	D147G, I154M	59	481

a CC, clonal complex.

b The primary sequence of each cluster is shown. For clusters 2 to 6, amino acids which differ from those in cluster 1 are identified by bold underline. Variants are listed alphabetically and are defined by amino acid substitutions that differ from the primary cluster sequence.

c Bold font designates clonal complexes that are phylogenetically separated from other strains in the same FarR cluster. For contextual purposes, the total number of genomes in the PATRIC database is 21,424, of which the numbers (*n*) corresponding to specific multilocus sequence types (MLSTs) are ST30 (562), ST239 (537), ST5 (3,484), ST8 (3135), ST97 (236), ST395 (3), ST228 (64), ST45 (636), ST101 (21), ST93 (56), ST12 (65), ST20 (54), ST1 (630), ST188 (201), ST1148 (1), ST398 (1,314), ST22 (2431), ST425 (20), ST50 (10), ST130 (28), ST133 (65), ST121 (250), ST152 (89), ST350 (2), ST705 (0), ST707 (8), and ST59 (481).

d Number of protein accessions identical to the primary clade or intraclade variant sequences, as determined by BLASTP of each sequence versus S. aureus subsp. *aureus* nonredundant proteins.

The proteome level phylogenetic tree is broadly congruent with previous DNA-based analyses and provides insight into the evolution of FarR in concert with phylogenetic diversity. Clusters 1 and 2 were both FarR^M85^, while clusters 3 to 6 were FarR^I85^ (Fig. S1, Table 1). Cluster 3 appears to reflect the evolution of S. aureus as a species because the primary cluster 3 FarR matched 266 protein accessions, some of which occurred in ST93 S. aureus (Table 1); however, identical proteins also occurred in S. argenteus, S. schweitzeri, S. roterodami, and S. singaporensis (data not shown). Moreover, cluster 3a occurred exclusively in the *S. argenteus* outgroup at the bottom of the phylogenetic tree (Fig. 1B). The FarR clusters were variable in the extent to which their associated clonal complexes conform to phylogenetic divisions, with different extremes noted in clusters 5 and 6. Cluster 6 and its variants all populate a single phylogenetic branch, whereas cluster 5 strains are dispersed across the phylogenetic spectrum (Fig. 1B), which likely represents historical recombination events involving *farR*.

The primary cluster 2 FarR protein matched 12,894 protein accessions across 14 clonal complexes, comprising a large section of the phylogenetic spectrum (Table 1, Fig. 1B). Cluster 2 was exceptional in exhibiting 14 variants, most of which occurred in the same clonal complexes represented among primary cluster 2 strains. Two exceptions were 2f (CC395) and 2l (CC45 and CC198), adjacent to the *S. argenteus* outgroup. At the nucleotide level, cluster 2f from CC395 shared some polymorphisms with ST93 cluster 3 *farR* (Fig. S2); and, as with cluster 3 FarR, cluster 2f was also M85I (Table 1, Fig. S1). Among the other cluster 2 variants, we noted non-conserved amino acid substitutions within CC5 strains, as evidenced by clusters 2a FarR^E160G^ and 2b FarR^G166D^ and an amino acid duplication in cluster 2c FarR^E93EE^ (Table 1). These represent the emergence of FarR variants within CC5, independent of phylogenetic diversity.

### Multiple FarR variants occur in CC5 S. aureus.

Because our analysis did not reveal FarR^H121Y^ or FarR^C116R^ variants that were previously discovered through *in vitro* selection procedures (21, 22) we conducted homology searches with each primary FarR cluster protein to search for additional variants in the S. aureus protein database. Although no strains had FarR^C116R^, several had FarR^C116Y^, and FarR^P165L^ was also identified (Table 2). Metadata for CC5 FarR variant strains are provided in Table S2, while non-CC5 variants are described in Table S3. FarR^E93EE^ and FarR^E160G^ were exclusive to CC5, while FarR^C116Y^, FarR^P165L^, and FarR^G166D^ occurred in CC5 and one or more nonrelated CCs (Table 2, Tables S2 and S3). Among the non-CC5 variants, strains with FarR^C116Y^ were CC1 or CC97 from bovine hosts, while FarR^G166D^ strains were ST8 and CC97 from human and bovine sources, respectively (Table 2 and Table S3). Although FarR^H121Y^ did not occur in CC5, it appeared sporadically in CC1, CC22, CC97, CC8 and CC30 from human and bovine hosts (Table 2, Table S3). Therefore, FarR^H121Y^, which we discovered through *in vitro* selection for increased resistance to LA, has emerged sporadically across different clonal complexes but not in CC5, while all other variants occurred in CC5. We considered that the occurrence of FarR^H121Y^, FarR^C116Y^, and FarR^G166D^ in different clonal complexes could represent *farR* exchange through recombination. However, nucleotide sequence alignments revealed that each *farR* variant has polymorphisms that are specific to the clonal complex from which it was recovered (Fig. S3), consistent with each variant having emerged independently through point mutation in different clonal complexes.

**TABLE 2:** FarR variants that occur independently of clonal complex diversity^*a*^

Variant	No. of genomes	MLST/CC	Source
H121Y	8	CC1 (ST2922), CC97, CC8, ST30, CC22; (EMRSA-15)	Human (CC1, ST30, ST8, CC22) and bovine (CC97)
C116Y	19	CC1 (ST3117), CC97 (ST97, ST71, ST3221, ST3109, ST3173), CC8	Bovine mastitis infection and milk; UK, Switzerland, Ireland, Turkey, Chile
	16	ST5/CC5	HA-MRSA; US, Canada
E93EE	61	CC5 (ST5, ST764)	HA-MRSA; Japan, China, Thailand, Europe, US
E160G	82	ST5/CC5	USA100 HA-MRSA; Israel, US, UK, Australia, Europe
P165L	34	ST5/CC5	HA-MRSA; US, Canada, Egypt, UK, Europe
	2	ST15	Scotland; MSSA
G166D	5	ST8 and CC97	Human ST8 (US and Norway) and bovine CC97 (UK)
	5	ST5	HA-MRSA; US and Netherlands

a MLST, multilocus sequence type; CC, clonal complex; ST, sequence type; HA-MRSA, hospital-acquired MRSA; MSSA, methicillin-susceptible S. aureus.

### CC5 and CC97 exhibit a disproportionate frequency of FarR variants compared to CC8.

Our data indicated that CC5 S. aureus was exceptional in exhibiting numerous FarR variants, represented by FarR^E93EE^, FarR^C116Y^, FarR^E160G^, FarR^P165L^, and FarR^G166D^, which cumulatively accounted for 198 protein accessions. For an approximation of the frequency of FarR variants, this would represent 5.7% of the 3,477 ST5 S. aureus genomes annotated in the PATRIC database (Table 3). Moreover, while there are a comparable number of ST8 S. aureus genomes in the PATRIC database (*n* = 3,125), the FarR^H121Y^, FarR^C116Y^, and FarR^G166D^ variants which occurred in CC8 represented just 0.1% of ST8 genomes (Table 3). Strikingly, this same repertoire of FarR^H121Y^, FarR^C116Y^, and FarR^G166D^ variants accounted for 8.1% of ST97 genomes (Table 3). Although this metadata analysis is subject to caveats, including a much smaller denominator for ST97 genomes, our analysis suggests that FarR variants are overrepresented in CC5 and CC97 compared to CC8.

**TABLE 3:** Frequency of FarR variants in ST5 compared to other MLSTs

MLST^*a*^	No. of genomes^*b*^	FarR variants (*n*)	%Variants^*c*^
5	3,477	E93EE (61), C116Y (16), E160G (82), P165L (34), G166D (5)	5.7
97	234	C116Y (16), H121Y (3), G166D (3)	8.1
8	3,125	C116Y (1), H121Y (1), G166D (2)	0.1
1	631	C116Y (2), H121Y (1)	0.3
22	2,440	H121Y (2)	0.08

a MLST, multilocus sequence type.

b Number of genomes in S. aureus PATRIC database corresponding to each MLST.

c Numerator is the sum of all FarR variants, denominator is the number of genomes.

### *farR* variants are distributed across the S. aureus CC5 phylogeny.

Our data have identified multiple FarR variants within CC5 S. aureus. Importantly, CC5 MRSA encompass multiple clones associated with health care-associated infections in the Western hemisphere (26), including an early branching CC5-basal clade and CC5-I and CC5-II clades which emerged in the early 1970s and early 1960s, respectively, followed by expansion in the Western hemisphere (26). We therefore conducted a phylogenetic comparison of 119 CC5 strains with variant FarR proteins and 26 comparator CC5 reference genomes to assess the distribution of FarR variants within the CC5 phylogeny. Metadata for these strains are provided in Table S2, and a list of polymorphisms that distinguish each strain from the S. aureus strain JH1 reference genome is provided in Table S4. Although our analysis of FarR variation in association with genetic diversity noted that a specific FarR cluster can occur in phylogenetically distinct clonal complexes, which is suggestive of historic recombination events, no evidence of recombination involving *farR* was detected with ClonalFrameML (CFML); neither in the current analysis of 145 CC5 strains nor in a prior study of 598 CC5 strains (26). Thus, FarR variants within CC5 are most likely the result of independent point mutations.

**FIG 2:**
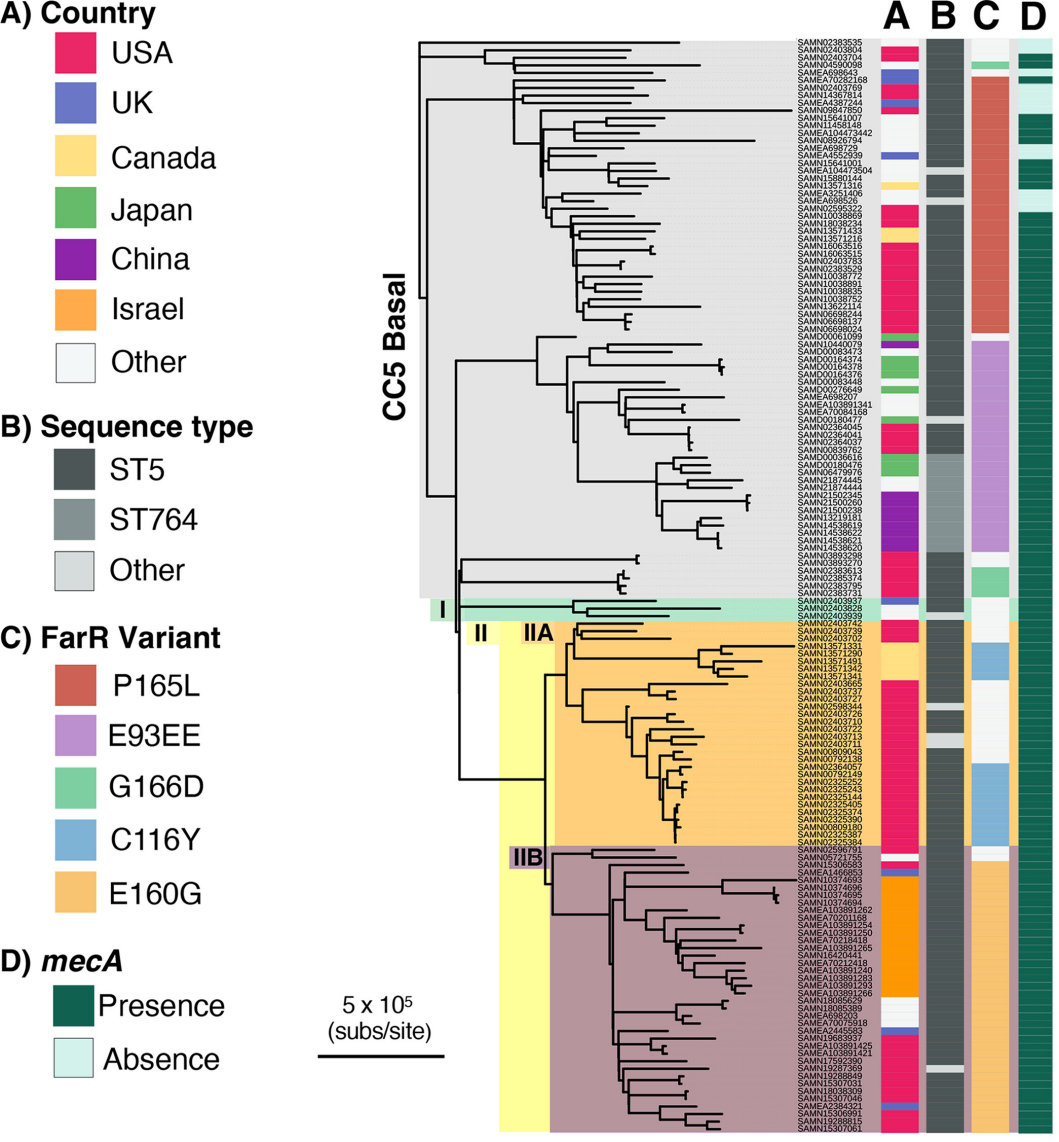
Distribution of *farR* variants within the representative worldwide S. aureus CC5 population. The phylogenetic distribution of strains with variant *farR* genes within the CC5 phylogeny was determined through a two-phase bioinformatics analysis. The first phase consisted of comparing polymorphisms in 119 CC5 strains with single amino acid substitutions in FarR to 598 CC5 reference strains (26), from which 26 reference genomes were selected that (i) subtended the nodes to which the new strains with FarR variants attached, (ii) provided examples of sister nodes of strains with FarR variants, and (iii) provided examples of the various CC5 clades previously defined. In the second phase, these 119 FarR variant strains and 26 reference genomes were analyzed with GATK to call SNPs and indels, as well as invariant core nucleotides relative to S. aureus JH1. The resulting bi-allelic SNPs and invariant core nucleotides were analyzed by PhyML and ClonalFrameML to generate a phylogeny and correct branch length for recombination. Here, strains with variant FarR proteins are placed in the context of CC5 phylogeny. The major CC5 clades, consisting of Basal, CC5-I, and CC5-II (-IIA and -IIB), as defined previously (26), are labeled on the root axis of the dendrogram. Biosample numbers from the NCBI genome sequence entries for each strain are shown adjacent to the branch structures. These strains are listed in the same order in Table S4, which also provides their common names (where available), SCC*mec* genotypes, and a list of polymorphisms for each strain relative to the S. aureus JH1 reference genome (83). Columns adjacent to the Biosample numbers provide information on country of origin (A), multilocus sequence type (MLST) designation (B), FarR variant (C), and presence or absence of *mecA* (D).

CC5 strains with variant FarR proteins were predominantly MRSA, since *mecA* was present in 25 of 34 strains with FarR^P165L^, 26 of 28 strains with FarR^E93EE^, and all strains with FarR^G166D^, FarR^C116Y^, or FarR^E160G^ (Fig. 2, Table S2). Strains with FarR^P165L^, FarR^G166D^, and FarR^E93EE^ emerged in the CC5-basal clade, while FarR^E160G^ and FarR^C116Y^ emerged in CC5-IIA and CC5-IIB, respectively (Fig. 2), which also contains the polyphyletic USA100 lineage of HA-MRSA that is endemic in North America (26, 27). Within the CC5-basal clade, strains with FarR^E93EE^ were most closely related to ST5 MRSA strain N315 from Japan (28), and most of these were also recovered from Japan, China, or Thailand (Fig. 2, Table S2). Conversely, FarR^P165L^ strains in CC5-basal are mainly from North America, but also occur in the United Kingdom, Europe, and Egypt. These strains are predominantly *spa* type t688 irrespective of their geographic location. FarR^G166D^ strains were polyphyletic, with one isolate from Europe being well separated from four FarR^G166D^ strains in the United States. These latter strains were most closely related to CC5 MRSA strains ISU979 and ISU936 recovered from swine (29). FarR^E160G^ strains emerged in CC5-IIA and were recovered primarily from the US and Israel, but also appeared in the United Kingdom, Europe, and Australia, and are predominantly *spa* t002, which is characteristic of the USA100 lineage of HA-MRSA. These strains were most closely related to a high-level vancomycin-resistant US strain VRS10 and strain UP109 from Peru, described as a multidrug-resistant strain belonging to the NY/Japan clone in the USA100 lineage (30, 31). FarR^C116Y^ strains within CC5-IIB are restricted to North America but, as with FarR^G166D^, are polyphyletic, with *spa* t002 strains from the United States being distinct from *spa* t5258 and *spa* t12967 strains from Canada (27). These two distinct emergences of FarR^C116Y^ strains are also most closely related to MRSA progenitors. Therefore, strains with variant *farR* genes have a global distribution and have emerged multiple times across the phylogenetic spectrum of CC5.

### Some FarR variant strains exhibit increased resistance to linoleic acid.

**FIG 3:**
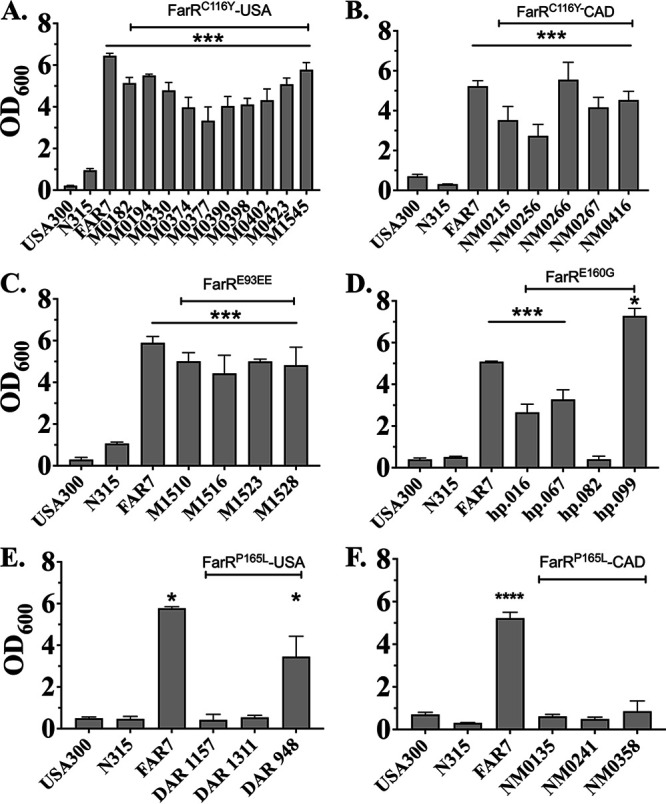
Graphs showing 24-h endpoint growth of USA100 FarR variants in tryptic soy broth (TSB) + 1,200 μM linoleic acid (LA). Cultures of USA300, N315, FAR7, or CC5 MRSA harboring either FarR^C116Y^ from the United States (A) or Canada (B), FarR^E93EE^ (C), FarR^E160G^ (D), or FarR^P165L^ from the United States (E) or Canada (F) were inoculated into triplicate tubes containing 3 mL of TSB + 1,200 μM LA + 0.1% dimethyl sulfoxide (DMSO) at an optical density at 600 nm (OD_600_) of 0.01, followed by incubation at 37°C with orbital shaking. Growth (OD_600_) was determined after 24 h. Each data point represents the mean ± standard deviation (SD) from triplicate cultures. Statistically significant differences (****, *P* < 0.0001; ***, *P* < 0.001; **, *P* < 0.01; *, *P* < 0.05) compared to N315 were determined by Tukey’s multiple-comparison test.

Although previous *in vitro* selection procedures for enhanced resistance to LA and rhodomyrtone led to the recovery of FarR^H121Y^ and FarR^C116R^ (21, 22) these did not occur in CC5. We therefore assessed the sensitivity of these strains to LA using the control strains USA300, the USA300-FarR^H121Y^ strain FAR7, and the ST5 MRSA reference strain N315 (28). Because FAR7 has an MIC of >1,200 μM LA (21), we chose FAR7 as a benchmark to assess resistance. USA300 and N315 exhibited negligible growth in 1,200 μM LA, while FAR7 exhibited good growth at a 24-h endpoint. All FarR^C116Y^ strains were resistant to 1,200 μM LA (Fig. 3A and B), as were four FarR^E93EE^ strains we obtained (Fig. 3C) and three out of four FarR^E160G^ variants (Fig. 3D). However, of six FarR^P165L^ strains from the United States and Canada, only the US strain DAR948 exhibited increased resistance (Fig. 3E and F).

**FIG 4:**
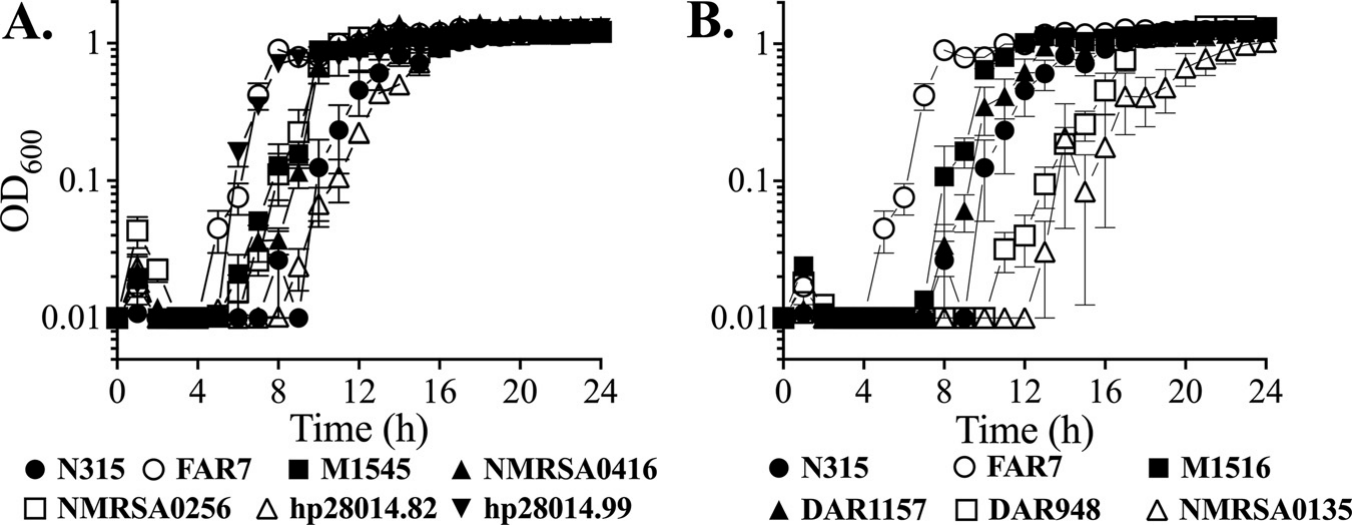
Growth of representative FarR variant strains in TSB + 200 μM LA. Cultures were inoculated to OD_600_ = 0.01 into 96-well microtiter plates containing 200 μL of TSB supplemented with a subinhibitory concentration of 200 μM LA + 0.1% DMSO. Plates were incubated at 37°C with orbital shaking, and growth (OD_600_) was monitored hourly. (A) FarR^C116Y^ (M1545, NM0256, NM0256) and FarR^E160G^ (hp.082 and hp.099) strains. (B) FarR^E93EE^ (M1516) and FarR^P165L^ (DAR1157, DAR948, NM0135) strains. Growth was compared to the ST-5 HA-MRSA (health care-associated MRSA) reference strain N315 and FAR7 (FarR^H121Y^). Each data point represents the mean ± standard error of the mean (SEM) from 6× 200-μL wells in 96-well microtiter plates.

Representative strains were also selected to assess growth in microtiter plates containing tryptic soy broth (TSB) + 200 μM LA, which is sub-MIC for USA300. Under these conditions, the FarR^H121Y^ strain FAR7 reached stationary phase in 7 h, at which time the ST5 HA-MRSA reference strain N315 was just emerging from an extended lag phase (Fig. 4A). The FarR^E160G^ strain hp20814.99, which was resistant to 1,200 μM LA, matched the growth of FAR7, while the single FarR^E160G^ strain hp20814.82 that was not resistant to 1,200 μM LA exhibited similar growth to that of N315. Strains representing the two occurrences of FarR^C116Y^ also exhibited enhanced growth compared to N315 (Fig. 4A), as did the FarR^E93EE^ strain M1516 (Fig. 4B). The FarR^P165L^ strains were variable, with DAR948 and NMRSA0315 showing delayed growth relative to N315, while the growth of DAR1157 was similar to that of N315 (Fig. 4B). These data mirror the outcomes from 24-h MIC endpoints, where FarR^C116Y^, FarR^E160G^, and FarR^E93EE^ strains which exhibited MICs of >1,200 μM LA also exhibited enhanced growth in TSB + 200 μM LA, while the FarR^P165L^ strains had no strong association with increased resistance.

### FarR^C116Y^ and FarR^E160G^ are sufficient to promote increased resistance to linoleic acid.

**FIG 5:**
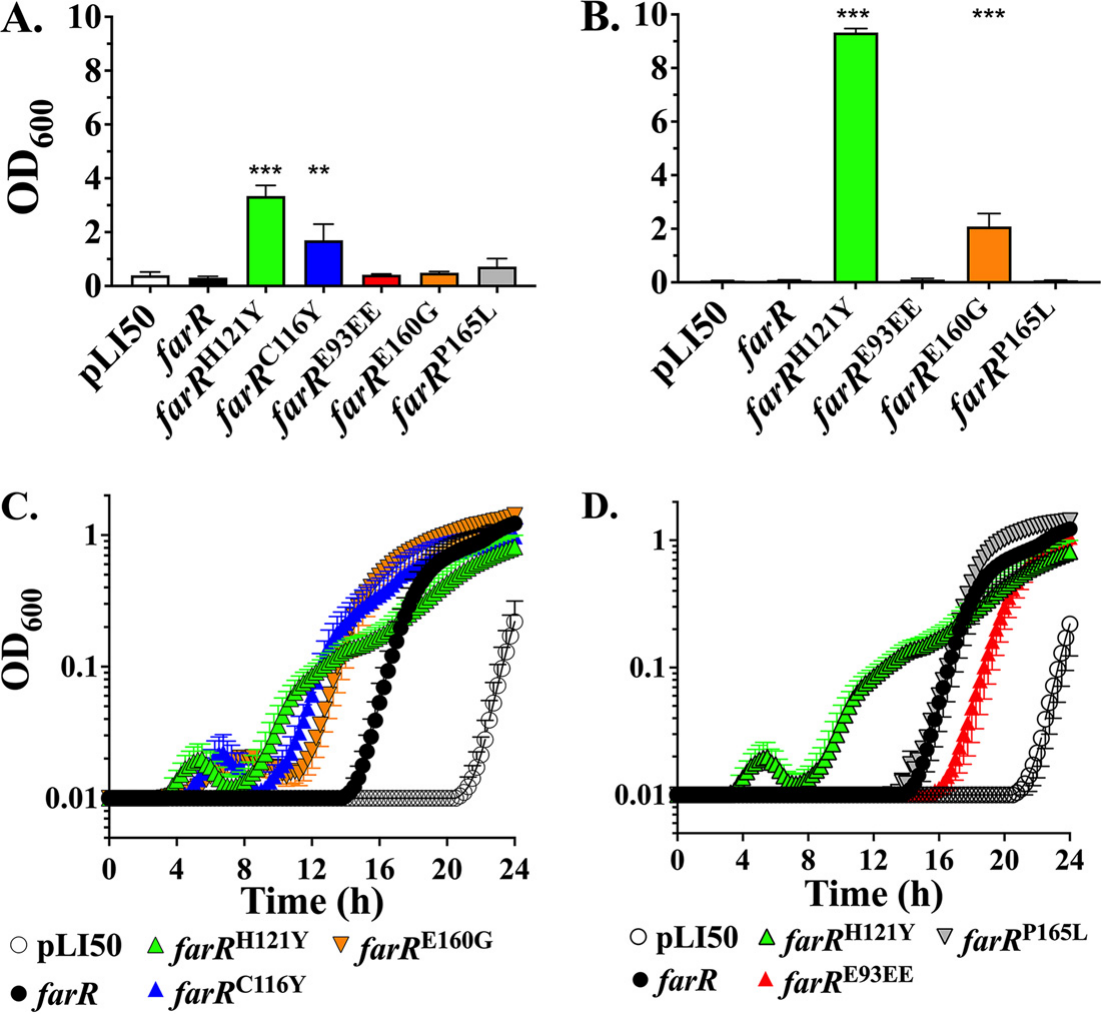
*farR*^C116Y^ and *farR*^E160G^ are sufficient to confer either increased resistance to, or growth advantage on exposure to, LA. Growth (OD_600_) of *farR*ΦNE harboring either pLI50 vehicle, pLI*farR*, or variants *farR*^H121Y^, *farR*^C116Y^, *farR*^E160G^, *farR*^P165L^, or *farR*^E93EE^ was assessed in TSB containing 1,200 μM (A) or 200 μM (B to D) LA + 0.1% DMSO. For panels A and B, endpoint growth (OD_600_) in culture tubes was assessed after 24 h, while panels C and D represent growth in microtiter plates with automated monitoring over 24 h. Each value represents the mean ± SD of triplicate 3-mL culture tubes (A and B) or the mean ± SEM of 6× 200-μL wells in 96-well microtiter plates (C and D). Statistically significant differences (***, *P* < 0.001; **, *P* < 0.01) compared to *farR*ΦNE + pLI*farR* wild type were determined by Tukey’s multiple-comparison test.

We previously demonstrated that complementation with plasmid pLI*farR*^H121Y^ was sufficient to promote enhanced LA resistance when transformed into strain *farR*ΦNE, in which *farR* is disrupted by a transposon insertion (21). We therefore constructed pLI*farR*^C116Y^, pLI*farR*^E160G^, pLI*farR*^P165L^, and pLI*farR*^E93EE^ to determine whether these variant genes confer enhanced resistance in *farR*ΦNE. As expected, pLI*farR*^H121Y^ conferred an MIC of >1,200 μM LA, as did pLI*farR*^C116Y^, but the other variants did not (Fig. 5A). However, when LA was reduced to 200 μM, pLI*farR*^E160G^ was able to confer growth at a 24-h endpoint (Fig. 5B). We therefore tested the ability of each variant to promote growth at a sub-MIC of 200 μM LA in 96-well microtiter plates. *farR*ΦNE + pLI50 vehicle exhibited a lag phase of more than 20 h, while pLI*farR* reduced the lag phase to ~14 h, which was further reduced to 4 to 5 h with pLI*farR*^H121Y^ or pLI*farR*^C116Y^ (Fig. 5C). The pLI*farR*^E160G^ construct also conferred a shorter lag phase relative to pLI*farR* (Fig. 5C), whereas pLI*farR*^P165L^ was indistinguishable from wild-type pLI*farR*, and pLI*farR*^E93EE^ conferred a longer lag phase (Fig. 5D). Therefore, we conclude that in addition to FarR^H121Y^, FarR^C116Y^ and, to a lesser extent, FarR^E160G^ are sufficient to confer increased LA resistance, whereas FarR^P165L^ and FarR^E93EE^ are not.

### Variant *farR* genes promote increased expression of *farE*.

**FIG 6:**
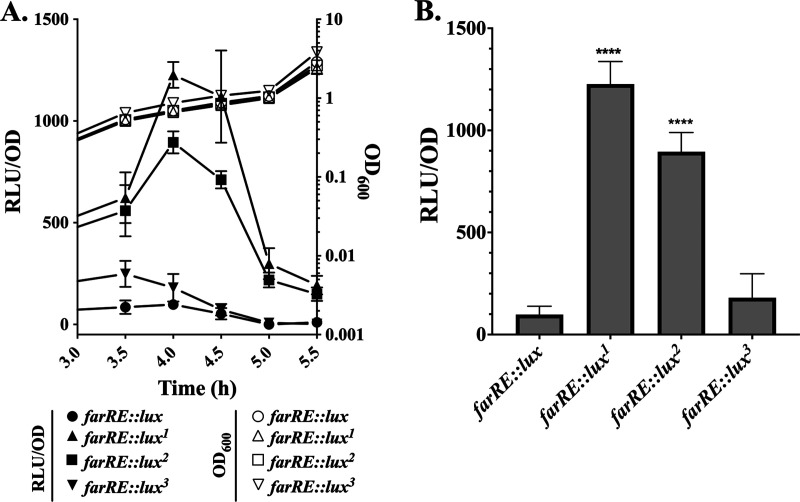
*farR* variants promote increased expression of *farE* in a *farRE*::*lux* luciferase reporter construct. *farR*ΦNE was transformed with either pGY*farRE*::*lux* or variant *lux*^1^ (*farR*^H121Y^), *lux*^2^ (*farR*^C116Y^), or *lux*^3^ (*farR*^E160G^) constructs where luciferase activity is driven from the P*_farE_* promoter under the control of wild-type or variant *farR* genes. Cultures were grown in 125-mL flasks containing 25 mL TSB for 3 h followed by supplementation with 40 μM LA + 0.1% DMSO. (A) Growth (OD_600_) and luciferase activity (relative light units [RLU]/OD_600_) was quantified at 30-min intervals. (B) Comparison of luciferase activity (RLU/OD_600_) at 1 h after addition of LA. Each data point represents the mean OD_600_ ± SD from triplicate flasks. Statistically significant differences (****, *P* < 0.0001) compared to wild-type *farER* were determined by Tukey’s multiple-comparison test.

To assess how variant *farR* alleles influence expression of *farE*, DNA segments containing *farR* and the adjacent P*_farE_* promoter were cloned in pGY*lux* such that luciferase expression is driven by P*_farE_* under the control of each *farR* variant. Cultures of S. aureus *farR*ΦNE containing these constructs were grown in TSB for 3h, followed by the addition of 40 μM LA and monitoring of luminescence at 30-min intervals. Cultures with *farRE*::*lux^1^* (FarR^H121Y^) and *farRE*::*lux^2^* (FarR^C116Y^) exhibited vastly elevated luminescence relative to wild-type *farRE*::*lux*, achieving a sharp peak after 1 h of exposure to LA and then rapidly receding (Fig. 6A and B). For *farRE*::*lux^3^* (FarR^E160G^), luminescence was increased relative to *farRE*::*lux* after 30 and 60 min, although this was not statistically significant. We therefore conclude that, as with FarR^H121Y^, FarR^C116Y^ and, to a lesser extent, FarR^E160G^ are also sufficient to confer increased LA resistance to S. aureus clinical isolates due to increased expression of the *farE* efflux pump.

### Phenotypic traits associated with pseudogenes and other polymorphisms in FarR^E93EE^ strains.

Because strains with FarR^E93EE^ were more resistant to LA through a mechanism which was not due to the variant *farR*, we conducted an analysis of how they differred from CC5 reference genomes and other variant FarR strains. Strains with FarR^E93EE^ were divided into branches A and B (Table 4, Fig. S4), with the larger B branch being subdivided such that B3 constitutes a new MLST designation, ST764. An accumulation of pseudogenes and other polymorphisms is also evident in the progression from A1 through to B3 (Table 4, Table S5).

**TABLE 4:** Pseudogenes and polymorphisms in FarR^E93EE^ variants^*a*^

Branch	Strains	Gene	Function	Defect
A and B	All except MC1031	JH1_0212	*uhpT* hexose phosphate uptake	fs@F301 (A1, A2); fs@I9 (B2, B3)
	All	JH1_2400	*lysR* family regulator	fs@I150
	All except SA-1B	JH1_2130	*ilvC* branched-chain amino acid synthesis	fs @E333
	All	JH1_0942	*lipA* lipoic acid synthase	Q302*
A1 and A2	KG-03, KG-18, KG-22, SA-1B, M1K003	JH1_0395	*glp*T glycerol-3-phosphate transporter	fs@S75; fs@S50
A1	SA-1B	**JH1_0994**	*lysR* family regulator	Y6*
A2	KG-03, KG-18, KG-22	JH1_2112	*agrA* response regulator	fs@F220
		JH1_2399	*hutU* histidine utilization	fs@K8
B2	All	JH1_0092	*araC* family regulator	fs@H82
		JH1_1680	*comE* competence protein	fs@K104
	M1523, M1528, M1510, M1516	JH1_0467	*hsdS* endonuclease	fs@K374
		JH1_0584	*fusA* elongation factor	V90A, L461S
		JH1_0586	M20 metallopeptidase	fs@A179
		JH1_0649	HAD family hydrolase	fs@K26
		**JH1_0998**	Phospholipid binding protein	S87*
		JH1_1275	*ylmH* RNA binding protein	fs@E213
		JH1_1800	Alanine dehydrogenase	fs@S340
	KUH180129	**JH1_0995**	*leuA*-like isopropylmalate synthase	fs@I24, K294
		**JH1_0997**	Membrane protein	fs@G214
B3	All	JH1_0842	LysE lysine/arginine export	fs@P95
		JH1_1456	Metallopeptidase	fs@G89
		JH1_2527	Carboxylesterase/lipase	W436*
		JH1_2610	*eamA* efflux pump	S204*
		JH1_2770	Sulfurtransferase	fs@H284
	SH-4, SH-3, SH-2	JH1_0584	*fusA* elongation factor	H457Q
	SH-4, SH-3, SH-2, M19, M153-2, M392, M212, M209	**JH1_0995**	*leuA*-like isopropylmalate synthase	fs@I78
	M19, M153-2, M392, M212, M209	JH1_0521	Methyltransferase	R66*
		JH1_1590	Short-chain dehydrogenase	fs@A143
		JH1_2133	*leuC* leucine synthesis	fs@A89
		JH1_2443	*hssR* heme response regulator	fs@A128
		JH1_2484	*fmhA* glycyltransferase	fs@K183
	M392, M212, M209	JH1_0393	*mepA* antimicrobial efflux	fs@S322
		JH1_2112	*agrA* response regulator	fs@E141

a Bold font indicates a series of tandem genes in which inactivating mutations have accrued.

All FarR^E93EE^ strains have a frameshift in SaurJH1_2490 encoding a LysR family regulator, which is adjacent to the *hutU* (JH1_2399) and *hutG* (JH1_2402) genes for histidine utilization. Branches A and B are distinguished by frameshifts at two sites in SaurJH1_0213 *uhpT* encoding a hexose phosphate transporter (Table 4 and Table S5), while Branch A strains also have a frameshift in *glpT* encoding a glycerol-3-phosphate transporter, and defects in these genes confer resistance to fosfomycin (32). Some strains in B2 and B3 have L461S or H457Q substitutions in FusA (Table 4), which confer resistance to fusidic acid (33, 34). Different strains in B2 and B3 also have frameshifts in co-associated genes SaurJH1_0995, JH1_0997, and JH1_0998, while strain SA-1B in branch A1 has a premature stop codon in a LysR-family regulator encoded by SaurJH1_0994. These genes, as depicted in Fig. S5, comprise an apparent operon that is divergently transcribed from the LysR regulator SaurJH1_0994. Strikingly, different strains in B2 and B3 have frameshifts within three poly(A) tracts in SaurJH1_0995, encoding the putative isopropylmalate synthase-like gene *leuA* (Fig. S5). Moreover, as with some of the FarR^E93EE^ strains, the most divergent branch of the FarR^P165L^ strains also exhibited a defect in the *leuA* ortholog encoded by JH1_0995 (Table S4; Fig. S5B), constituting a shared trait among some strains with FarR^P165L^ and FarR^E93EE^.

**FIG 7:**
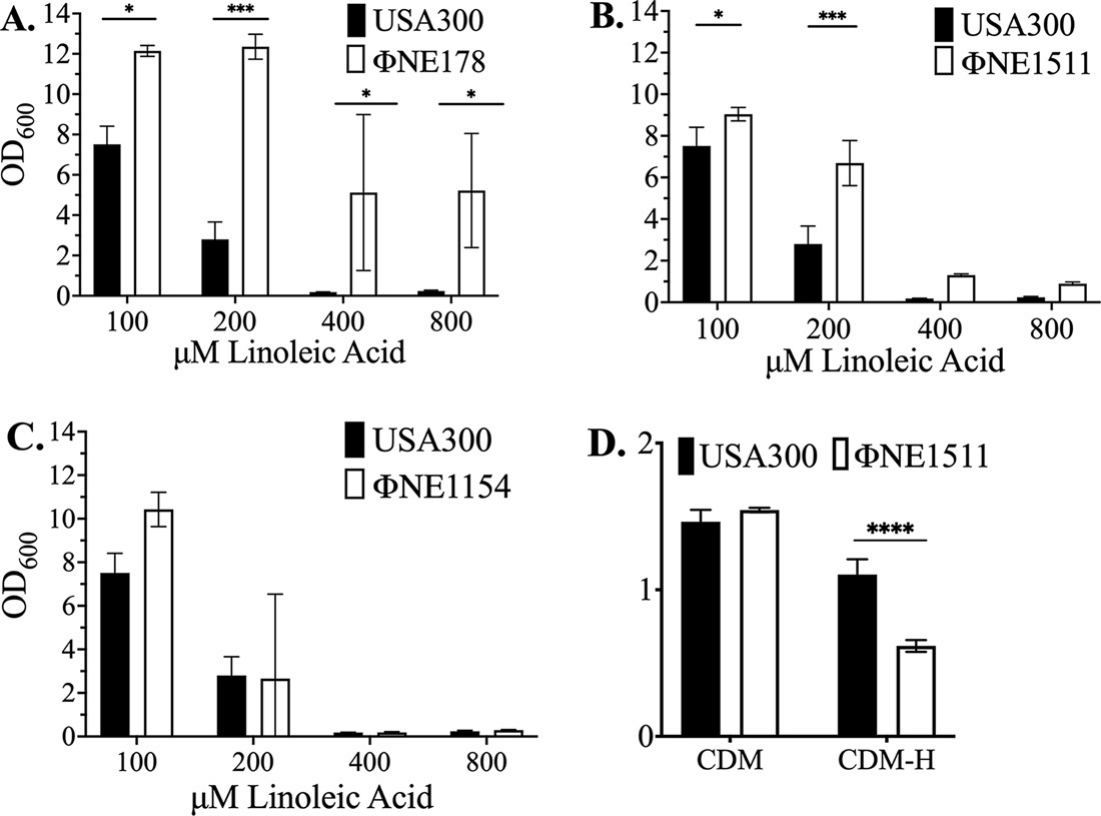
Resistance and growth phenotypes of USA300 harboring transposon insertions in genes that exhibit frameshift mutations in FarR^E93EE^ strains. Cultures of USA300 or isogenic variants ΦΝΕ178 (A), ΦΝΕ1511 (B), or ΦΝΕ1154 (C), with transposon insertions in *leuA*, *lysR*, or *uhpT*, respectively, were grown in TSB containing the indicated concentrations of LA + 0.1% DMSO. Each datapoint represents the mean OD_600_ ± SD from triplicate 3-mL tube cultures after 24 h growth. (D) Growth of USA300 and isogenic ΦΝΕ1511 (*lysR*::*tn*) in chemically defined medium containing 0.4% glucose (CDM-G) or 0.25% histidine (CDM-H) as a carbon source. All data points represent the mean ± SD from triplicate 3-mL tube cultures after 24 h growth. Statistically significant differences (****, *P* < 0.0001; ***, *P* < 0.001; *, *P* < 0.05) compared to S. aureus USA300 were determined by Tukey’s multiple-comparison test.

In view of these observations, we queried whether loss of gene function could influence resistance to LA using the Nebraska transposon mutant library in S. aureus USA300 (35). USA300ΦΝΕ178 with a transposon insertion in the *leuA*-like isopropylmalate synthase exhibited increased resistance to LA (Fig. 7A). For inactivation of the *lysR* regulator of histidine metabolism in ΦΝΕ1511, enhanced growth was conferred in 100 and 200 μM LA, but the MIC was not altered (Fig. 7B), whereas inactivation of *uhpT* in ΦΝΕ1154 did not significantly alter growth or the MIC (Fig. 7C). Because the function of *lysR* as a regulator of histidine metabolism has not previously been addressed, we also assessed the growth of USA300 and ΦΝΕ1511 in a chemically defined medium (CDM) with either glucose (CDM-G) or histidine (CDM-H) as a carbon source. Both strains grew well in CDM with glucose, but ΦΝΕ1511 growth was significantly impaired in CDM-H, where glucose was replaced with additional histidine (Fig. 7D).

With a frameshift in *uhpT*, FarR^E93EE^ strains should be deficient in using hexose-6-phosphate as a carbon source and resistant to fosfomycin. Accordingly, the growth of FarR^E93EE^ strain M1516 was significantly impaired in CDM-G6P (CDM supplemented with glucose-6-phosphate) compared to that in CDM, whereas the ST5 HA-MRSA reference strain N315 and a FarR^C116Y^ strain M182, both with a functional *uhpT*, did not exhibit impaired growth in CDM-G6P (Fig. S6A). Strain M1516 was also resistant to fosfomycin, as predicted (Fig. S6B).

## DISCUSSION

We discovered FarR and the divergently transcribed efflux pump FarE through *in vitro* selection for increased resistance to LA (21, 36), an antimicrobial uFFA that would be encountered at sites of colonization and infection. This selected for FarR^H121Y^ in S. aureus USA300, promoting increased expression of FarE and enhanced resistance to LA. We have now established that this same variant has emerged in S. aureus strains of diverse genetic backgrounds from both human and bovine hosts, but it did not appear to expand within any of these genetic backgrounds and it was not detected among CC5. Conversely, all other FarR variants occurred within CC5 and exhibited a broad geographic distribution. Of these, FarR^C116Y^ and FarR^E160G^ were individually sufficient to promote increased resistance to LA. Others have noted that *in vitro* selection for resistance to the lipopeptide antibiotic rhodomyrtone led to recovery of FarR^C116R^ in a S. aureus lab strain HG001 (22). We did not detect this variant in any S. aureus strains, whereas FarR^C116Y^ has emerged twice within CC5 MRSA, and in CC1 and CC97 strains from bovine sources. In addressing the mechanism of enhanced resistance to rhodomyrtone, it was found that highly elevated FarE expression promoted the release of membrane phospholipid (37). Consequently, mutations that promote extremely high FarE expression could exert a deleterious phenotype, which may account for the failure of strains with FarR^H121Y^ to expand within any genetic background, while strains with FarR^C116R^ were not detected in any S. aureus clinical isolates.

Over the past 2 decades, ST5 MRSA strains have been among the most common clones causing hospital-acquired infections in the Western hemisphere (26). This is also the principal genetic background for the emergence of high-level vancomycin resistance through the acquisition of novel d-ala ligases, as well as a common background for an intermediate resistance phenotype through the accumulation of point mutations (30, 38). However, CC5 is rivaled by CC8 for prominence of MRSA in the Western hemisphere, and with the emergence of the ST8 USA300 strain of CA-MRSA, evolutionary trends and phylogenetics of ST8 MRSA have also been extensively studied (39, 40) such that if FarR variants have become established within ST8 MRSA, they should have been found in our analysis. This underscores the remarkable adaptive capacity of ST5 MRSA, which, in addition to multiple antimicrobial resistance genes, now includes the emergence of variant FarR proteins.

Here, we have established that FarR^C116Y^ and FarR^E160G^ alone are sufficient to confer increased resistance to LA. It is not known whether the emergence of these variants can be attributed to exposure to antibiotics or elevated concentrations of antimicrobial fatty acids in certain niche environments. However, different *in vitro* selection procedures using either LA or the lipopeptide rhodomyrtone both led to the recovery of FarR variants with increased resistance (21, 22), while selection for increased resistance to an oxadiazole antibiotic promoted the recovery of a variant FarE protein (23). Exposure to antibiotics in animal-based agricultural practices promotes the emergence of resistance (41): the non-CC5 FarR^C116Y^ and FarR^H121Y^ variants were recovered from bovine isolates on dairy farms, while FarR^G166D^ variants from the United States are most closely related to strains from porcine sources (29). Moreover, strains with FarR^C116Y^, FarR^E160G^, FarR^E93EE^, and FarR^G166D^ all appear to have emerged within CC5 MRSA, such that repeated exposure of MRSA to antibiotics and host-derived antimicrobial fatty acids in the context of nosocomial infections could be a driving force in the emergence of FarR variants in CC5 MRSA.

It remains to be determined whether variant FarR proteins confer a competitive advantage within a particular host niche. However, the occurrence of FarR^H12IY^, FarR^C116Y^, and FarR^G166D^ strains within CC1 and CC97 bovine hosts is suggestive of selection for the emergence of such variants in other settings besides human infections, where successful strains would be repeatedly exposed to antibiotics and host-derived antimicrobial fatty acids. Notably, bovine mastitis is often chronic, and FarR variant proteins could confer an advantage in chronic infections because LA is the most abundant antimicrobial fatty acid in tissue abscess homogenates (42). In the context of chronic infections, FarR^E160G^ variants were recovered from patients with infectious endocarditis and cystic fibrosis (43, 44). This includes a study that assessed the evolution of antibiotic resistance in MRSA bacteremia that persisted for at least 2 weeks despite antimicrobial therapy (44). Only 2 of 48 patients conformed to these criteria, and both had infective endocarditis. From our analysis, it is evident that in both cases, the index MRSA at the onset of bacteremia were FarR^E160G^ strains. However, we also note that FarR^P165L^ strains were recovered from patients with cystic fibrosis at different locations in the United States (45, 46), and this variant is not sufficient to confer increased resistance to LA. As such, it is feasible that some FarR variants may promote phenotypes that have yet to be elucidated.

Although four strains that we obtained with FarR^E93EE^ exhibited increased resistance to LA, we could not attribute this to the variant FarR. Other Far^E93EE^ strains we identified included KG-03, which progressed to a vancomycin-intermediate resistance phenotype over the course of prolonged bacteremia therapy (47). Our data also support a trend toward a multiple-resistance phenotype in FarR^E93EE^ strains because all of these have a defect in *uhpT*, while some are also defective in *glpT*, and these mutations promote resistance to fosfomycin (32). Fosfomycin has been evaluated as a combination therapy with daptomycin for treatment of MRSA bacteremia with endocarditis (32, 48, 49); this practice could contribute to greater future prevalence of these strains. Some FarR^E93EE^ strains also have amino acid substitutions in FusA that confer resistance to the topical antimicrobial agent fusidic acid, including three FusA^H457Q^ strains with high-level resistance (33), and four with FusA^L461S^ that we obtained for this study.

Although we were not able to precisely define the mechanism by which FarR^E93EE^ strains have increased resistance toward LA, our analysis documents an accumulation of pseudogenes, a trait associated with niche adaptation (50–53). The effects of the pseudogenes include restricted access to carbon sources due to defects in LysR, a regulator of histidine metabolism, as well as *uhpT* and *glpT*, which promote uptake of glucose-6-phosphate and glycerol-3-phosphate, respectively. A four-gene operon beginning with SaurJH1_0995, encoding a *leuA*-like isopropylmalate synthase, was also a target of inactivating mutations in different FarR^E93EE^ strains. Although the FarR^E93EE^ strains we obtained did not have defects in this gene, USA300 ΦΝΕ178, in which this gene (SAUSA300_0879) is disrupted by a transposon insertion, exhibited a significant increase in the MIC for LA, which supports our contention that increased LA resistance can occur through adaptive mechanisms.

As with FarR^E93EE^, FarR^P165L^ was insufficient to confer increased resistance to LA. Nevertheless, these strains exhibited a broad geographic distribution, being recovered from the United States, Egypt, Denmark, Canada, and the United Kingdom. Most of these were *spa* t688, including MRSA from patients with cystic fibrosis at two locations in the United States (45, 46), and one MRSA recovered from cheese in Egypt (54). There are also several reports of ST5 *spa* t688 MRSA recovered from livestock and food sources, in addition to hospital settings (55–60), primarily in Egypt, Kuwait, Algeria, and Italy. Therefore, a future assessment of the co-association of FarR^P165L^ with *spa* t688 MRSA is warranted.

In summary, we have defined the emergence and spread of S. aureus strains with variant FarR proteins in CC5 MRSA, of which FarR^C116Y^ and FarR^E160G^ confer increased resistance to LA. FarR^C116Y^ variants emerged twice within the CC5 phylogeny but were also frequently recovered in CC97 strains from bovine hosts. In contrast, FarR^E160G^ was unique to CC5, and some of these strains were recovered from patients with infective endocarditis and cystic fibrosis (44, 61). Future questions to address include whether such variants are more common in these patient populations and how these amino acid substitutions contribute to increased expression of FarE. TetR family regulators normally repress expression of RND superfamily efflux pumps such that enhanced efflux-mediated resistance is achieved through mutations which inactivate the TetR repressor. However, in S. aureus, FarE cannot be expressed in the absence of FarR (21, 36), and because a common trait of TetR family regulators is that their affinity for DNA is modulated by binding small molecule ligands (62), it is possible that variant amino acids modify the ligand-binding function to promote enhanced expression of FarE. Future work will address these considerations.

## MATERIALS AND METHODS

### Cultures, plasmids, and growth conditions.

S. aureus and Escherichia coli strains and plasmids that were constructed for this study and variant FarR strains obtained for this study are listed in Table 5. The S. aureus clinical isolates with variant FarR proteins (FarR^E93EE^, FarR^C116Y^, FarR^E160G^, and FarR^P165L^) were obtained from collections maintained by the Brigham and Women’s Hospital (Massachusetts Microbiome Center), Anthony Fisher at the University of Iowa Medical Center (43), George Golding with the Canadian Nosocomial Infection Surveillance Program and the Public Health Agency of Canada (27), and Ashley Robinson at University of Mississippi Medical Center (26, 40). A list of S. aureus genomes with variant *farR* genes and ST5 reference genomes used for phylogenetic construction is provided in Table S1. S. aureus and E. coli were cultured in TSB and LB, respectively, containing 15 g/L agar when required for growth on solid medium. Cultures were maintained at −80°C in 20% glycerol; when required for experimental purposes, cultures from frozen stocks were streaked on agar medium and single colonies were inoculated into polypropylene tubes containing 3 mL of the appropriate broth and grown overnight at 37°C on an orbital shaker at 220 rpm. When necessary for plasmid maintenance, TSB was supplemented with 5 μg/mL chloramphenicol or 3 μg/mL erythromycin, and LB was supplemented with 100 μg/mL ampicillin. When inoculum was needed for experimental purposes, single colonies were inoculated into 3 mL of the appropriate broth in polypropylene snap-cap tubes and were grown overnight at 37°C, rotating at 220 rpm on an orbital shaker.

**TABLE 5:** S. aureus and E. coli strains and plasmids^*a*^

Strain or plasmid	Description	Reference or Biosample ID
S. aureus		
RN4220	Restriction endonuclease deficient strain capable of accepting foreign DNA (*r_k_*– *m_k_+*)	64
USA300	CA-MRSA, wild-type strain cured of resistance plasmids	86, 87
N315	ST5 HA-MRSA	88
FAR7	*in vitro*-selected FarR^H121Y^ variant of USA300; increased resistance to linoleic acid	21
*farR*ΦNE	Transposon insertion in *farR* (SAUSA300_2490); Nebraska library mutant ΝΕ1393 was transduced into plasmid-cured USA300; Erm^R^	21, 35
*farR*ΦNE pLI50	*farR*ΦNE with pLI50 complementation vector; Cm^r^, Erm^r^	21
*farR*ΦNE pGY*lux*	*farR*ΦNE with pGY*lux* luciferase reporter vector; Cm^r^, Erm^r^	21
*farR*ΦNE pLI*farR^xy^*	*farR*ΦNE complemented with *farR^xy^* cloned in pLI50; *farR^xy^* segment derived from USA300, FAR7 (*farR^H121Y^*), and ST5 MRSA with *farR^C116Y^*, *farR^E93EE^*, *farR^E160G^*, or *farR^P165L^* variants; Cm^r^, Erm^r^	21
*farR*ΦNE pGY*farRE::lux^x^*	*farR*ΦNE with pGY*lux* vector containing *farR^xy^* gene and P*_farE_* promoter segment driving luciferase reporter expression; *farR^xy^E* segment derived from USA300 (*farRE*::*lux*), FAR7 (*farR^H121Y^*; *farRE*::*lux*^1^), and ST5 MRSA with *farR^C116Y^* (*farRE*::*lux*^2^) or *farR^E160G^* (*farRE*::*lux*^3^) Cm^r^, Erm^r^	This study
ΝΕ1154	USA Nebraska transposon mutant library strain, containing a transposon insertion in *uhpT* (SAUSA300_0216). Transposon insertion was confirmed by PCR with primers NE1154_F and NE1154_R (Table 5); Erm^r^	35
ΝΕ1511	USA300 Nebraska transposon mutant library strain containing a transposon insertion in putative *lysR* regulator of histidine metabolism (SAUSA300_2279). Transposon insertion was confirmed by PCR with primers NE1511_F and NE1511_R (Table 5); Erm^r^	35
ΝΕ178	USA300 Nebraska transposon mutant library strain containing a transposon insertion in a *leuA*-like isopropylmalate synthase (SAUSA300_0879). Transposon insertion was confirmed by PCR with primers NE178_F and NE178_R (Table 5); Erm^r^	35
M0182	FarR^C116Y^ ST5 HA-MRSA	SAMN02325243
M0194	FarR^C116Y^ ST5 HA-MRSA	SAMN02325252
M0330	FarR^C116Y^ ST5 HA-MRSA	SAMN00792149
M0374	FarR^C116Y^ ST5 HA-MRSA	SAMN00809180
M0377	FarR^C116Y^ ST5 HA-MRSA	SAMN02325374
M0390	FarR^C116Y^ ST5 HA-MRSA	SAMN02325384
M0398	FarR^C116Y^ST5 HA-MRSA	SAMN02325387
M0402	FarR^C116Y^ ST5 HA-MRSA	SAMN02325390
M0423	FarR^C116Y^ ST5 HA-MRSA	SAMN02325405
M1545	FarR^C116Y^ ST5 HA-MRSA	SAMN02364057
NMRSA0256	FarR^C116Y^ ST5 HA-MRSA	27
NMRSA0215	FarR^C116Y^ ST5 HA-MRSA	27
NMRSA0416	FarR^C116Y^ ST5 HA-MRSA	27
NMRSA0267	FarR^C116Y^ ST5 HA-MRSA	27
NMRSA0266	FarR^C116Y^ ST5 HA-MRSA	27
M1510	FarR^E93EE^ ST5 HA-MRSA	SAMN00839762
M1516	FarR^E93EE^ ST5 HA-MRSA	SAMN02364037
M1523	FarR^E93EE^ ST5 HA-MRSA	SAMN02364041
M1528	FarR^E93EE^ ST5 HA-MRSA	SAMN02364045
hp20814.016	FarR^E160G^ ST5 HA-MRSA	61
hp20814.067	FarR^E160G^ ST5 HA-MRSA	61
hp20814.082	FarR^E160G^ ST5 HA-MRSA	61
hp20814.099	FarR^E160G^ ST5 HA-MRSA	61
DAR 1157	FarR^P165L^ ST5 HA-MRSA	26
DAR 1311	FarR^P165L^ ST5 HA-MRSA	26
DAR 948	FarR^P165L^ ST5 HA-MRSA	26
NMRSA0241	FarR^P165L^ ST5 HA-MRSA	27
NMRSA0358	FarR^P165L^ ST5 HA-MRSA	27
NMRSA0135	FarR^P165L^ ST5 HA-MRSA	27
E. coli		
DH5α	Transformation competent strain. λ^−^ϕ80d*lacZ*ΔM15 Δ(*lacZYA*-*argF*)*U169 recA1 endA1 hsdR17(r_K_− m_K_−) supE44 thi-1 gyrA relA1*	Invitrogen
Plasmid		
pLI50	E. coli–S. aureus shuttle vector; Amp^r^, Cm^r^	89
pLI*farR*	pLI50 with native *farR* gene; Amp^r^, Cm^r^	21
pLI*farR^H121Y^*	pLI50 with *farR^H121Y^* from S. aureus FAR7; Amp^r^, Cm^r^	21
pLI*farR^C116Y^*	pLI50 with *farR*^C116Y^ from S. aureus M1545; Amp^r^, Cm^r^	This study
pLI*farR^E93EE^*	pLI50 with *farR*^E93EE^ from S. aureus M1516; Amp^r^, Cm^r^	This study
pLI*farR^E160G^*	pLI50 with *farR*^E160G^ from S. aureus hp20814.099; Amp^r^, Cm^r^	This study
pLI*farR^P165L^*	pLI50 with *farR*^P165L^ from S. aureus DAR 948; Amp^r^, Cm^r^	This study
pGY*lux*	E. coli–S. aureus shuttle vector harboring promoterless *lux*ABCDE operon; Amp^r^, Cm^r^	66
pGY*farRE*::*lux*	pGY*lux* with native *farR* and P*_farE_* promoter segment from USA300 cloned in pGY*lux*; Amp^r^, Cm^r^	This study
pGY*farRE*::*lux^1^*	pGY*lux* with *farR* and P*_farE_* promoter segment from S. aureus FAR7 (H121Y) cloned in pGY*lux*; Amp^r^, Cm^r^	This study
pGY*farRE*::*lux^2^*	pGY*lux* with *farR* and P*_farE_* promoter segment from S. aureus M1545 (C116Y) cloned in pGY*lux*; Amp^r^, Cm^r^	This study
pGY*farRE*::*lux^3^*	pGY*lux* with *farR* and P*_farE_* promoter segment from S. aureus hp20814.99 (E160G) cloned in pGY*lux*; Amp^r^, Cm^r^	This study

a CA-MRSA, community-acquired MRSA; HA-MRSA, hospital-acquired MRSA.

For growth assays in chemically defined medium containing 0.4% glucose as the carbon source, CDM-G was prepared as described previously (63) from 10× stock solutions of defined l-amino acids (alanine, arginine, aspartate, cysteine, glutamate, glycine, histidine, isoleucine, leucine, lysine, methionine, phenylalanine, proline, serine, threonine, tryptophan, tyrosine, and valine), vitamins (l-thiamine, nicotinic and pantothenic acid, biotin), salts (K_2_HPO_4_ and KH_2_PO_4_), and divalent cations (MgSO_4_, [NH_4_]_2_SO_4_, MgCl_2_, and CaCl_2_). Where indicated, CDM-G was modified by the omission of glucose and the addition of either 0.25% glucose-6-phosphate (CDM-G6P) or 0.25% l-histidine (CDM-His) as a carbon source.

### Strain and plasmid construction.

Genetic manipulation of S. aureus was conducted following established guidelines (64) and as described in our previous work (21, 36, 65). Restriction enzymes and T4 DNA ligase were purchased from New England BioLabs, *Taq* polymerase from GenScript, kits for PCR cleanup and plasmid preparation from Geneaid, and nucleotide primers (Table 6) from Integrated DNA Technologies. All plasmids were constructed as shuttle vectors in E. coli DH5α and their integrity was confirmed by nucleotide sequencing of the cloned DNA fragments, using the primers pLI50_F and pLI50_R for genes cloned in pLI50, and pGY*lux*_F and pGY*lux*_R for products cloned in pGY*lux*. All shuttle vectors were then transformed by electroporation into S. aureus USA300 or isogenic derivatives using S. aureus RN4220 as an intermediate host. The PCR primers *farR_*F1 and *farR*_R1 were used to PCR-amplify variant *farR* genes from S. aureus clinical isolates, which were then digested with KpnI and SacI for cloning in pLI50 to create complementation plasmids in which *farR* is expressed from its native promoter as previously described for wild-type *farR* and *farR*^H121Y^ (21). For luciferase reporter constructs, primers *farRE*::*lux*_F and *farRE*::*lux*_R were used to amplify a 905-bp product comprising the *farR* gene and adjacent P*_farE_* promoter segment, which was then digested with BamHI and SalI for ligation into the pGY*lux* reporter vector (66).

**TABLE 6:** PCR primer sequences

Primer	Sequence^*a*^
pLI50_F	5'–ATTTCCCCGAAAAGTGCC-3'
pLI50_R	5'–TTTCTCGGCATAAATGCG-3'
pGYlux_F	5'–CTGTTGTTTGTCGGTGAACGCT-3'
pGYlux_R	5'–ATTGGGGAGGTTGGTATGTAAGC-3'
*farR*_F1	5'–cccggtaccTGCAGCTACAATCACTATCCATGC-3'
*farR*_R1	5'–cccgagctcACGGACGCTAAAACAGGTAGTCC-3'
*farRE*::*lux*_F	5'–CGATAGTAGTACACGgATcCATTAACGTGTACACTATCG-3'
*farRE*::*lux*_R	5'–CATTGTCAAATgTCGacGCATTTGTAGCAAGTGG-3'
NE178_F	5'–GGCATGTGTACAACTATCGAGG-3'
NE178_R	5'–AACATCCTACAGTGTCCGCA-3'
NE1511_F	5'–TCGAAAGCACCATTCCGACT-3'
NE1511_R	5'–AGTGTTTGCGCACTTGAGAAT-3'
NE1154_F	5'–TTAAGCGCTTACACCGACGT-3'
NE1154_R	5'–AAGTATCGGCCACGTTTCGT-3'

a Underlined lowercase nucleotides represent 5- additions for incorporation of KpnI or SacI restriction sites (*farR*_F1 and *farR*_R1, respectively) or primer-directed changes to the template sequence for incorporation of BamHI or SalI restriction sites (*farRE*::*lux*_F and *farRE*::*lux*_R, respectively).

### Analysis of FarR variation in the context of S. aureus phylogenetic diversity.

We considered all available complete S. aureus genomes, and an *S. argenteus* genome to serve as an outgroup, from the Pathosystems Resource Integration Center (PATRIC) database as of 2020 (25). Genomes that were of low quality based on various considerations or high divergence based on mash distance were removed from consideration. Phylogenetic analysis of the S. aureus proteome, represented by conceptually translated nucleotide sequences available from PATRIC, was performed for 574 S. aureus and 1 *S. argenteus* strains using PhyloPhlAn3 (67). Proteins were mapped to an S. aureus reference database using Usearch (68), aligned with Muscle (69), and gappy regions in the alignment were trimmed with Trimal (70), resulting in a curated, concatenated alignment of 21,922 variable sites. Initial phylogenetic analysis used FastTree, which was refined with RaxML under the PROTCATLG model (71). 563 full-length FarR proteins from these strains were then extracted from the PATRIC database, and a minimum spanning tree was constructed by alignment of the FarR amino-acid sequences using GrapeTree (72). This identified six primary FarR sequence clusters that were mapped onto the strain phylogeny.

### Identification of FarR variant proteins.

Different *in vitro* selection procedures promoted recovery of FarR^H121Y^ and FarR^C116R^ variants. As such, variants that are candidates for altering FarR function were likely to be identified through amino acid substitutions that distinguished FarR in a specific strain, from other S. aureus strains within the same clonal complex, as noted within some CC5 strains which were identified through analysis of variation across 574 genomes in the PATRIC database. For a complete analysis, each of the six primary FarR cluster sequences was used in a BLASTP search (73) to assess variation in FarR across all S. aureus genome assemblies, as well as the additional assemblies described below, to identify amino acid substitution variants that occur within specific genetic backgrounds. Additional FarR variants were identified from Sequence Read Archive (SRA) data, including MRSA from hospitals in Ontario, Canada (27) and additional strains from Europe. For this purpose, sequence data from all isolates was used to generate *de novo* assemblies with Spades v.3.12.0 (74). The adapters and low-quality reads were removed with Cutadapt v1.16 (75) and Sickle v1.33 (76) and the contamination screen was completed using FastQ Screen (77). Optimal k-mers were identified based on average read lengths for each genome. All assemblies were evaluated using QUAST v.5.0.1 (78). The reads were mapped back to *de novo* assemblies to investigate polymorphism (which is indicative of mixed cultures) using Bowtie2 v1.2.2 (79). Low-quality genome assemblies were removed from further analysis (i.e., *N*_50_ < 10,000, total assembly length outside the median sequence length of ±1 standard deviation [SD], contigs smaller than 1 kb contributing to >10% of the total assembly length).

### Genotyping and phylogenetic analyses.

Multilocus sequence typing was done by scanning genome assemblies against the S. aureus MLST database (https://pubmlst.org/saureus/) using the mlst v2.10 program with default settings (https://github.com/tseemann/mlst). Detection of *mecA* and typing of SCC*mec* was done by mapping the strains’ pseudoreads to a custom-clustered database of *ccr* and *mec* gene complexes, plus SCC*mec* IV subtype-specific sequences (80), using SRST2 v0.2.0 (81) with the min_coverage 60 option. Staphylococcal protein A gene (*spa*) typing was conducted using spaTyper (https://spatyper.fortinbras.us/).

The placement of FarR variant strains within the CC5 phylogeny was assessed in a two-phase approach. First, genome assemblies from 119 strains with single amino acid substitutions in FarR (FarR^E93EE^, FarR^C116Y^, FarR^E160G^, FarRP^165L^, FarRG^166D^) were confirmed as CC5 using multilocus sequence typing. A total of 250,000 PE pseudoreads (100 bp each, 200-bp fragment size) were produced from these assemblies using samtools wgsim, mapped to the S. aureus JH1 reference genome with bwa, and finally realigned around indels with GATK as performed previously (26). Mapped nucleotide sites were exported as a VCF file with the ‘emit all sites’ option in GATK. Using this file, a lookup was done on the positions of 11,961 biallelic single-nucleotide polymorphisms (SNPs) identified from the 598 CC5 reference strains in the study of Challagundla et al. (26) to assign the 119 strains’ alleles at those SNPs. The SNP data from these 119 new strains were combined with those from the 598 reference strains, giving a total of 717 strains. A BioNJ tree was generated using the total number of SNP differences as a distance, and the tree was rooted with a previously identified outgroup strain, DAR3176 (26). This tree was used to select 30 reference strains which (i) subtended the nodes to which the new strains with FarR variants attached, (ii) provided examples of sister nodes of strains with FarR variants, and (iii) provided examples of the various CC5 clades previously defined.

For the second phase, a final analysis of the 117 FarR variant strains and the 30 selected reference strains was done with GATK Unified Genotyper (82) to call SNPs and indels, as well as invariant core nucleotide sites relative to the reference genome S. aureus JH1 (83). Sites present in all samples with 3 or more reads per sample were retained. This resulted in an alignment of 9,020 bi-allelic SNPs and 2,292,396 invariant core nucleotide sites that was analyzed with PhyML (HKY+G+I) (84) and then with ClonalFrameML (85) to generate a phylogeny and correct its’ branch lengths for recombination. This tree places the strains with FarR variants in the context of the CC5 phylogeny and is shown in Fig. 2.

### Growth and MIC assays.

To prepare the inoculum for growth assays, single colonies of S. aureus clinical isolates were inoculated into 3 mL TSB in 12 × 75-mm polypropylene snap-cap tubes and grown overnight at 37°C on a rotary shaker. The optical density at 600 nm (OD_600_) of these overnight cultures was determined on a Beckman-Coulter DU 530 Spectrophotometer. For MIC determination with LA, cells were subcultured to OD_600_ = 0.01 in triplicate 20 × 150-mm glass tubes with plastic caps containing 3 mL TSB + 0.1% DMSO (dimethyl sulfoxide) and the indicated concentrations of LA. Tubes were placed on a rack at a 30° angle in a 37°C incubator with shaking at 220 rpm, and OD_600_ was measured after 24 h. For growth assays, cultures were inoculated into 96-well microtiter plates containing 200 μL TSB + 0.1% DMSO and the indicated concentrations of LA. The growth of each culture was assessed in 6 wells. Plates were incubated at 37°C on a rotary shaker (220 rpm) and OD_600_ was monitored at hourly intervals using a Synergy H4 Hybrid Reader (BioTek, Winooski, VT); alternately, where indicated, the plates were incubated at 37°C in the Synergy H4 Reader with automated shaking and optical density measurements taken every 15 min for 24 h. For growth in CDM-G, CDM-G6P, and CDH-His, 3-mL inoculum cultures grown overnight in the respective CDM media were subcultured to an OD_600_ of 0.01 into 3 mL of the appropriate medium in 20 × 150-mm glass tubes and grown as described for MIC determination, with endpoint growth (OD_600_) measured at 24 h.

### Sensi-disc antibiotic sensitivity assay.

To test for sensitivity or resistance to fosfomycin, S. aureus cultures were grown overnight in TSB, then diluted to OD_600_ = 0.01 in fresh sterile TSB. This was used to saturate a sterile Q-tip swab, which was then used to swab the surface of a tryptic soy agar plate, followed by addition of a BBL Sensi-disc (Becton, Dickinson and Co.) containing fosfomycin (200 μg). Plates were incubated at 37°C for 18 h and photographed to document zones of inhibition.

### Assays of reporter gene expression.

Overnight inoculum cultures containing the appropriate reporter constructs were subcultured to OD_600_ = 0.01 into triplicate 125-mL flasks containing 25 mL of TSB + 0.1% DMSO, and then incubated at 37°C with orbital shaking (220 rpm) for 3 h, at which point the cultures were supplemented by addition of 40 μM LA. Samples (4× 200-μL aliquots) were withdrawn at this time point (*t* = 0), and again at 30-min intervals, into 96-well white opaque flat-bottomed plates (Greiner Bio-One). Wells were supplemented with 20 μL of 0.1% (vol/vol) decanal in 40% ethanol and luminescence measurements were immediately taken on a Synergy H4 hybrid reader (BioTek), with 1 s of integration and a gain of 200. Synchronously, OD_600_ was measured in triplicate with a spectrophotometer. Data values were recorded as relative light units (RLU), corrected for background by subtracting values recorded from cultures harboring empty pGY*lux*. Data points were standardized for differences in growth by dividing RLU values by the recorded OD_600_ values of the cultures when samples were withdrawn.
