# Supplementary material for: Repeated Emergence of Variant TetR Family Regulator, FarR, and Increased Resistance to Antimicrobial Unsaturated Fatty Acid among Clonal Complex 5 Methicillin-Resistant Staphylococcus aureus
Source: Antimicrob Agents Chemother. 2023 Feb 6;67(3):e00749-22. doi: 10.1128/aac.00749-22 (PMC10019231; doi:10.1128/aac.00749-22)
Supplement: Supplemental file 3 — Fig. S1 to S6. Download aac.00749-22-s0003.pdf, PDF file, 5.4 MB [file aac.00749-22-s0003.pdf]

Figure S1

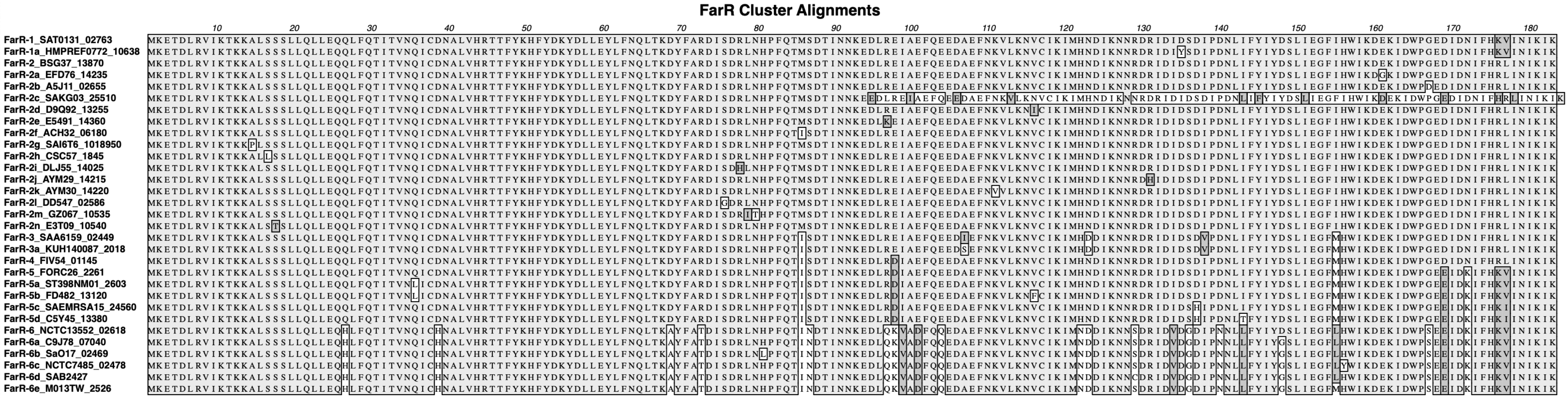
FarR Cluster Alignments
FarR-1_SAT0131_02763
FarR-1a_HMPREF0772_10638
FarR-2_BSG37_13870
FarR-2a_EFD76_14235
FarR-2b_A5J11_02655
FarR-2c_SAKG03_25510
FarR-2d_D9Q92_13255
FarR-2e_E5491_14360
FarR-2f_ACH32_06180
FarR-2g_SAI6T6_1018950
FarR-2h_CSC57_1845
FarR-2i_DLJ55_14025
FarR-2j_AYM29_14215
FarR-2k_AYM30_14220
FarR-2l_DD547_02586
FarR-2m_GZ067_10535
FarR-2n_E3T09_10540
FarR-3_SAA6159_02449
FarR-3a_KUH140087_2018
FarR-4_FIV54_01145
FarR-5_FORC26_2261
FarR-5a_ST398NM01_2603
FarR-5b_FD482_13120
FarR-5c_SAEMRSA15_24560
FarR-5d_C5Y45_13380
FarR-6_NCTC13552_02618
FarR-6a_C9J78_07040
FarR-6b_SaO17_02469
FarR-6c_NCTC7485_02478
FarR-6d_SAB2427
FarR-6e_M013TW_2526

Figure S2

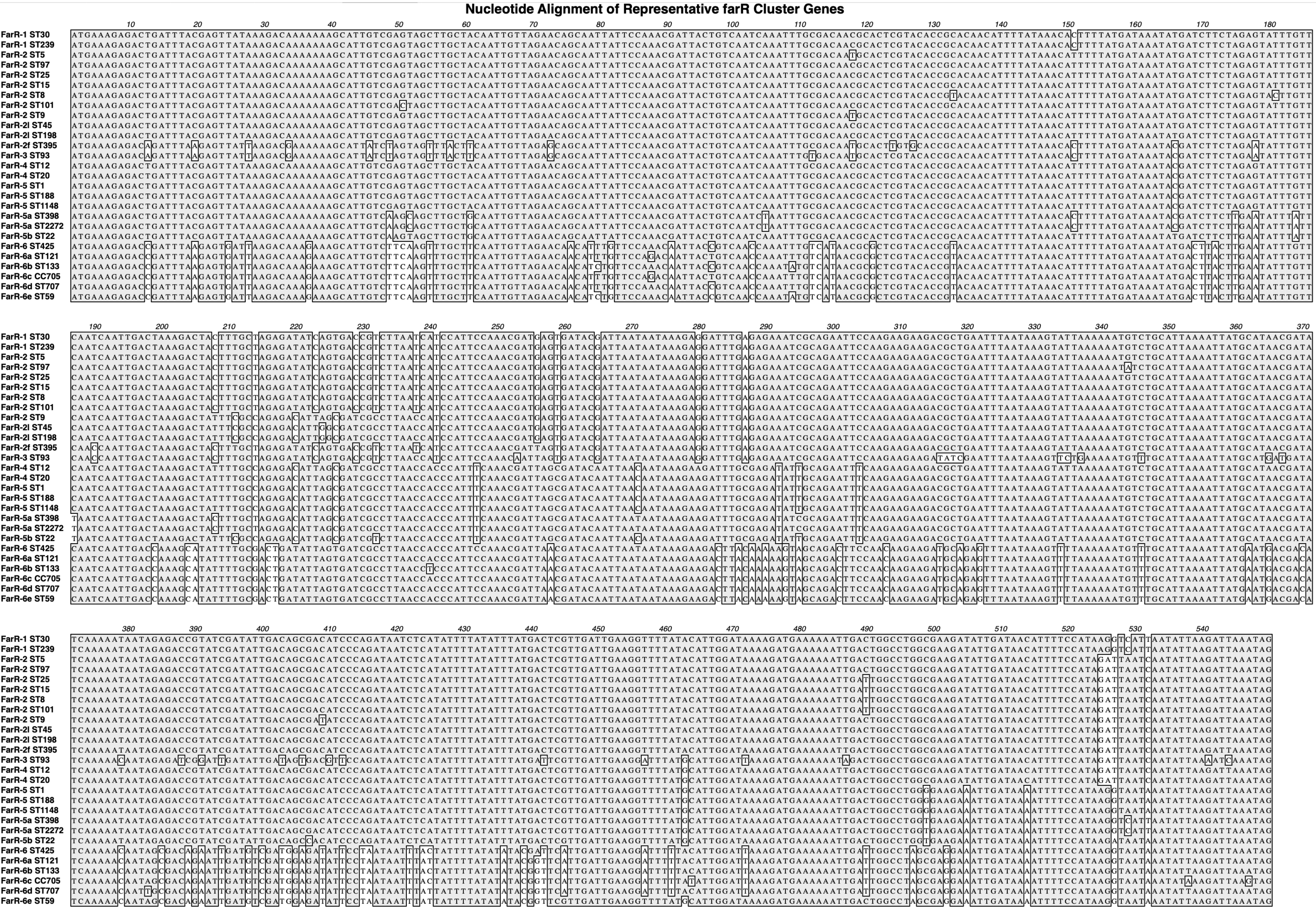

Nucleotide Alignment of Representative farR Cluster Genes
FarR-1 ST30
FarR-1 ST239
FarR-2 ST5
FarR-2 ST97
FarR-2 ST25
FarR-2 ST15
FarR-2 ST8
FarR-2 ST101
FarR-2 ST9
FarR-2l ST45
FarR-2l ST198
FarR-2f ST395
FarR-3 ST93
FarR-4 ST12
FarR-4 ST20
FarR-5 ST1
FarR-5 ST188
FarR-5 ST1148
FarR-5a ST398
FarR-5a ST2272
FarR-5b ST22
FarR-6 ST425
FarR-6a ST121
FarR-6b ST133
FarR-6c CC705
FarR-6d ST707
FarR-6e ST59

Figure S3

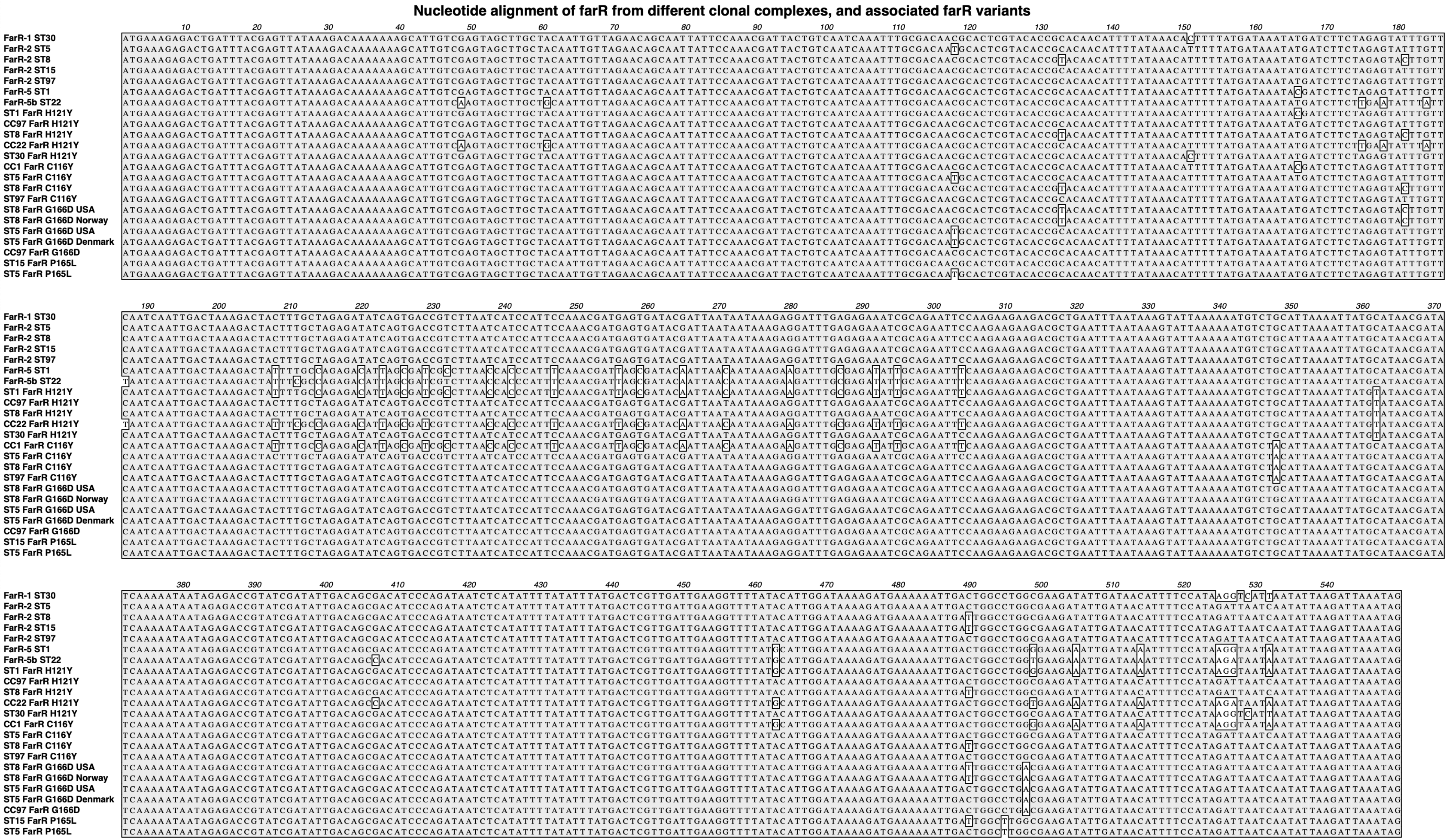
Nucleotide alignment of farR from different clonal complexes, and associated farR variants
FarR-1 ST30
FarR-2 ST5
FarR-2 ST8
FarR-2 ST15
FarR-2 ST97
FarR-5 ST1
FarR-5b ST22
ST1 FarR H121Y
CC97 FarR H121Y
ST8 FarR H121Y
CC22 FarR H121Y
ST30 FarR H121Y
CC1 FarR C116Y
ST5 FarR C116Y
ST8 FarR C116Y
ST97 FarR C116Y
ST8 FarR G166D USA
ST8 FarR G166D Norway
ST5 FarR G166D USA
ST5 FarR G166D Denmark
CC97 FarR G166D
ST15 FarR P165L
ST5 FarR P165L

Figure S4: Phylogenetic detail of cluster 2c (FarR-E93EE) strains.

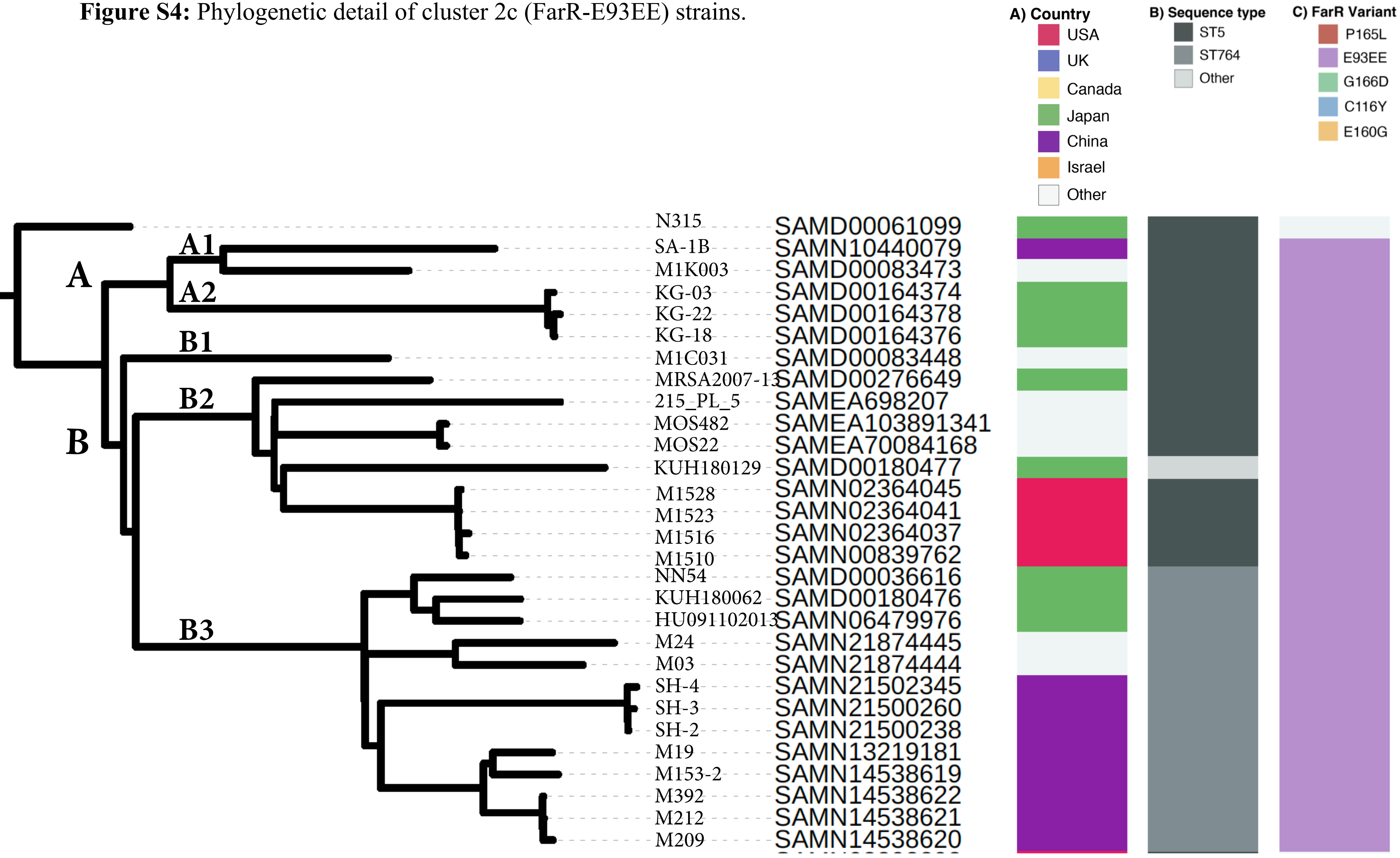

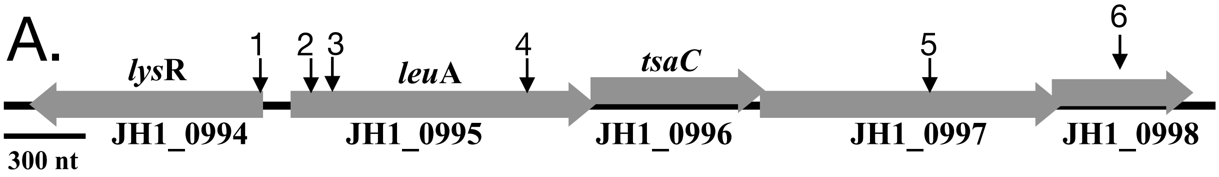

B.

ATGATTCGAATTCAAGATAATACAATAAGGGATGGTATGCAACAAAGTAATGTTCGAAAAAGTCTAA
TTAT**AAAAAAA**GAAGTATTGAAACAAATTAACAAGTTAAATATAAATTCTGTTGAAGTAGGCATGTG
TACAACTATCGAGGATGAATTTAATATTCATCAATTCAGAGACATTTTAAGTCCTGAAAAAGAATTA
GTAGTATTGACTAGGCTTAATGAAAAAGAAAT**AAAAAAAA**TAGTCAAATTGAAAATTCATAATTTAG
TGGTAAAAATACTATTGCCAATATCTGACTTGCATATAAAAGAAAAGCTTAATTTTTCAAATAAATA
TTATATTCAGAAAATCAAAGACTGCTTGGATATATTAAAGAAAGATAAAAAAGGAGTAGATATTTGT
TTTGAAGATGCAACAAGGACTTCTAGAGAAAAATTGAAAGAATACATGGAAATTATTTCAAAATATC
AAGTTAGAACAGTTACATTTGCGGACACTGTAGGATGTTCGACACCATTAGAATACGGAGATATTTT
TAATTACTTTGTAAAAAAATATTCTAACATAATTTTTTCTGCTCATTGTCATAACGATCTAGGGTTG
GCTACTGCAAATACATTAGCTGCAATTTTAAATGGTGCAAAGCAAATAGAAACTACATTTTTGGGAA
TTGGTGAGAGAGCGGGTAATGCTCCTATTGAGGAAATAATTACTATTTTGAC***AAAAAAA***CAAATAGA
AAGTACGGAATTCACTTTACCCGACGTATATAAAACTAGTATTAATATTTCTAAAATTCTCGATTTT
CAAATATCAGAAAACAAACCTATAATTGGTGAAAATATATTTAAACATGAATCAGGAATTCATCAAG
ATGGTACT**AAAAAAAA**TATAAATATGTATCAATATTTAGTTCCTAGTGATTTAGGATTGAAAAATTC
ACAAGTTGTTCAGTTTCCAATAAGTAATATTTCTAGTAAGAAAATCTTGCACAATAAATTTAAATCA
ATAGTTAACACTGAAGAAATTGATGAAAATATTTCTTTCTATAAACTTGTGAACAAGTTTTCACCTG
AAGTAGCACCTGAAGATACAGTGGATTTACTTCAGATAATAAAAAGGAGGAGTAAAGATGGAAATTT
TAAATGA

Fig. S5A: *lysR-leuA* locus and accumulated pseudogenes in FarR^{E93EE} strains. *lys*R, LysR family transcriptional regulator; *leu*A, putative isopropylmalate synthase; *tsaC*, putative threonylcarbomyladenylate synthase; JH1_0997, membrane associated protein; JH1_0998, putative phospholipid binding protein. The location of single nucleotide polymorphisms that confer pseudogenes in different strains are indicated by arrows; **(1)**, Y6* premature stop codon in SA-1B; **(2)**, frameshift at codon I24 in KUH180129; **(3)**, frameshift at I78 in SH-4, SH-3, SH-2, M19, M153-2, M392, M212, and M209; **(4)**, frameshift at K294 in KUH180129; **(5)**, frameshift at G214 in KUH180129; **(6)**, frameshift at S87 in M1510, M1516, M1523, and M1528.

Fig. S5B: Nucleotide sequence and location of indels at polyA segments in *leuA* (SaurJH1_0995). **Bold underlined** segments represent sites 2, 3 and 4 respectively in S5A, and the ***bold italic*** segment indicates the site of a frameshift in some FarR^{P165L} strains.

A.

B.

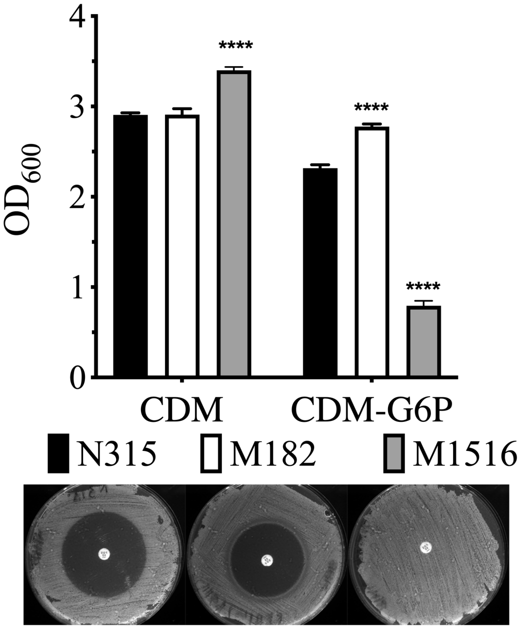

Figure S6. Phenotype of FarR^{E93EE} strain M1516 is consistent with a defect in *uhpT*.

(A), Growth of *S. aureus* strains N315, M182 (FarR^{C116Y}) and M1516 (FarR^{E93EE}) in CDM containing glucose or glucose-6-phosphate (G6P) as carbon source. All data points represent the mean ± SD from triplicate 3 mL tube cultures after 24h growth. Statistically significant differences (****, P<0.0001; ***, P<0.001; *, P<0.05) compared to *S. aureus* N315 were determined by Tukey's multiple comparison test. **(B),** Fosfomycin sensi-disc assay. A Fosfomycin sensi-disc (BBL) containing 200 µg fosfomycin was placed on the surface of a TSA plate that had been swabbed with *S. aureus* N315, M182 or M1516. Plates were photographed after incubation at 37ºC for 18h.
